# Genome-wide Association Meta-analysis of Childhood and Adolescent Internalizing Symptoms

**DOI:** 10.1016/j.jaac.2021.11.035

**Published:** 2022-04-01

**Authors:** Eshim S. Jami, Anke R. Hammerschlag, Hill F. Ip, Andrea G. Allegrini, Beben Benyamin, Richard Border, Elizabeth W. Diemer, Chang Jiang, Ville Karhunen, Yi Lu, Qing Lu, Travis T. Mallard, Pashupati P. Mishra, Ilja M. Nolte, Teemu Palviainen, Roseann E. Peterson, Hannah M. Sallis, Andrey A. Shabalin, Ashley E. Tate, Elisabeth Thiering, Natàlia Vilor-Tejedor, Carol Wang, Ang Zhou, Daniel E. Adkins, Silvia Alemany, Helga Ask, Qi Chen, Robin P. Corley, Erik A. Ehli, Luke M. Evans, Alexandra Havdahl, Fiona A. Hagenbeek, Christian Hakulinen, Anjali K. Henders, Jouke Jan Hottenga, Tellervo Korhonen, Abdullah Mamun, Shelby Marrington, Alexander Neumann, Kaili Rimfeld, Fernando Rivadeneira, Judy L. Silberg, Catharina E. van Beijsterveldt, Eero Vuoksimaa, Alyce M. Whipp, Xiaoran Tong, Ole A. Andreassen, Dorret I. Boomsma, Sandra A. Brown, S. Alexandra Burt, William Copeland, Danielle M. Dick, K. Paige Harden, Kathleen Mullan Harris, Catharina A. Hartman, Joachim Heinrich, John K. Hewitt, Christian Hopfer, Elina Hypponen, Marjo-Riitta Jarvelin, Jaakko Kaprio, Liisa Keltikangas-Järvinen, Kelly L. Klump, Kenneth Krauter, Ralf Kuja-Halkola, Henrik Larsson, Terho Lehtimäki, Paul Lichtenstein, Sebastian Lundström, Hermine H. Maes, Per Magnus, Marcus R. Munafò, Jake M. Najman, Pål R. Njølstad, Albertine J. Oldehinkel, Craig E. Pennell, Robert Plomin, Ted Reichborn-Kjennerud, Chandra Reynolds, Richard J. Rose, Andrew Smolen, Harold Snieder, Michael Stallings, Marie Standl, Jordi Sunyer, Henning Tiemeier, Sally J. Wadsworth, Tamara L. Wall, Andrew J.O. Whitehouse, Gail M. Williams, Eivind Ystrøm, Michel G. Nivard, Meike Bartels, Christel M. Middeldorp

**Affiliations:** Mss. Jami and Hagenbeek, Mr. Ip, and Drs. Hammerschlag, Hottenga, Beijsterveldt, Boomsma, Nivard, Bartels, and Middeldorp are with Vrije Universiteit Amsterdam, Amsterdam, the Netherlands. Ms. Jami and Dr. Allegrini are with University College London, London, United Kingdom. Drs. Hammerschlag, Hagenbeek, and Bartels are also with Amsterdam Public Health Research Institute, Amsterdam, the Netherlands. Drs. Hammerschlag and Middeldorp are also with the Child Health Research Centre, University of Queensland, Brisbane, Australia. Dr. Middeldorp is also with the Child and Youth Mental Health Service, Children’s Health Queensland Hospital and Health Service, Brisbane, Australia. Dr. Allegrini, Rimfeld, and Plomin are with the Social, Genetic and Developmental Psychiatry Centre, King’s College London, London, United Kingdom. Drs. Benyamin, Zhou, and Hypponen are with the University of South Australia, Adelaide, Australia. Drs. Benyamin and Hypponen are also with South Australian Health and Medical Research Institute, Adelaide, Australia. Drs. Border, Corley, Evans, Hewitt, Smolen, Stallings, and Wadsworth are with the Institute for Behavioral Genetics, University of Colorado Boulder. Drs. Diemer, Vilor-Tejedor, Neumann, Rivadeneira, and Tiemeier are with Erasmus University Medical Center, Rotterdam, the Netherlands. Dr. Neumann is also with the Lady Davis Institute for Medical Research, Jewish General Hospital, Montreal, Canada. Drs. Diemer, Vilor-Tejedor and Tiemeier are with Harvard T.H. Chan School of Public Health, Boston, Massachusetts. Mr. Jiang and Drs. Q. Lu, Tong, Burt, and Klump are with Michigan State University, East Lansing. Mr. Jiang and Dr. Tong are also with the University of Florida, Gainesville. Dr. Karhunen is with Imperial College London, United Kingdom. Drs. Y. Lu, Chen, Kuja-Halkola, Larsson, Lichtenstein, and Ms. Tate are with Karolinska Institutet, Stockholm, Sweden. Mr. Mallard and Dr. Harden are with the University of Texas, Austin. Drs. Pashupati and Lehtimäki are with Tampere University, Tampere, Finland, and Fimlab Laboratories, Tampere, Finland. Drs. Nolte, Hartman, Oldehinkel, and Snieder are with the University of Groningen, University Medical Center Groningen, the Netherlands. Mr. Palviainen, Drs. Korhonen, Vuoksimaa, Kaprio, and Ms. Whipp are with the Institute for Molecular Medicine Finland - FIMM, University of Helsinki, Finland. Drs. Peterson, Silber, and Maes are with Virginia Institute for Psychiatric and Behavioral Genetics, Virginia Commonwealth University, Richmond. Dr. Maes is also with Massey Cancer Center, Virginia Commonwealth University, Richmond. Drs. Sallis and Munafò are with the School of Psychological Science, University of Bristol, United Kingdom, and Medical Research Council (MRC) Integrative Epidemiology Unit, University of Bristol, United Kingdom. Dr. Sallis is also with the Centre for Academic Mental Health, Population Health Sciences, University of Bristol, United Kingdom. Dr. Munafo is also with NIHR Biomedical Research Centre at the University Hospitals Bristol NHS Foundation Trust and the University of Bristol, United Kingdom. Drs. Shabalin and Adkins are with the University of Utah, Salt Lake City. Drs. Thiering, Heinrich, and Standl are with the Institute of Epidemiology, Helmholtz Zentrum Munchen - German Research Center for Environmental Health, Neuherberg, Germany. Drs. Thiering and Heinrich are also with the Ludwig-Maximilians-Universität, Munich, Germany. Dr. Heinrich is also with the Melbourne School of Population and Global Health, The University of Melbourne, Victoria, Australia. Dr. Vilor-Tejedor is with the Erasmus University Medical Center, Rotterdam, the Netherlands; Centre for Genomic Regulation (CRG), The Barcelona Institute of Science and Technology, Barcelona, Spain; BarcelonaBeta Brain Research Center, (BBRC) Pasqual Maragall Foundation, Barcelona, Spain; and Universitat Pompeu Fabra (UPF), Barcelona, Spain. Dr. Vilor-Tejedor, Alemany, and Sunyer are with the Universitat Pompeu Fabra (UPF), Barcelona, Spain. Drs. Alemany and Sunyer are also with SGlobal, Barcelona Institute of Global Health, Barcelona, Spain; and CIBER Epidemiologıa y Salud Publica (CIBERESP), Spain. Dr. Sunyer is also with IMIM (Hospital del Mar Medical Research Institute), Barcelona, Spain. Ms. Wang and Dr. Pennell is with the School of Medicine and Public Health, University of Newcastle, Australia. Drs. Ask, Havdahl, Reichborn-Kjennerud, and Ystrøm are with the Norwegian Institute of Public Health, Oslo, Norway. Dr. Ystrøm is also with PROMENTA Research Center, University of Oslo, Norway. Dr. Ehli is with Avera Institute for Human Genetics, Avera McKennan Hospital & University Health Center, Sioux Falls, South Dakota. Drs. Hakulinen and Keltikangas- Järvinen are with the University of Helsinki, Helsinki, Finland. Ms. Henders is with the Institute for Molecular Biosciences, University of Queensland, Brisbane, Australia. Dr. Mamun is with the Institute for Social Science Research, University of Queensland, Brisbane, Australia. Ms. Marrington and Drs. Najman and Williams are with the School of Public Health, University of Queensland, Brisbane, Australia. Dr. Andreassen is with NORMENT Centre, Institute of Clinical Medicine, University of Oslo, Oslo, Norway; and Oslo University Hospital, Norway. Drs. Brown and Wall are with the University of California San Diego, La Jolla. Dr. Copeland is with the University of Vermont, Burlington. Dr. Dick is with Virginia Commonwealth University, Richmond. Dr. Harris is with the Carolina Population Center, University of North Carolina at Chapel Hill. Dr. Hopfer is with the University of Colorado, Aurora. Dr. Jarvelin is with MRC-PHE Centre for Environment and Health, Imperial College London, United Kingdom; the Center for Life Course Health Research, University of Oulu, Oulu, Finland; and Oulu University Hospital, Oulu, Finland. Dr. Krauter is with the University of Colorado Boulder. Dr. Lundstrom is with the University of Gothenburg, Swe-€ den. Dr. Magnus is with the Centre for Fertility and Health, Norwegian Institute of Public Health, Oslo, Norway. Dr. Njølstad is with the Center for Diabetes Research, University of Bergen, Bergen, Norway, and Haukeland University Hospital, Bergen, Norway. Drs. Reynolds and Rose are with the University of California at Riverside, California, and Indiana University, Bloomington, Indiana. Dr. Whitehouse is with Telethon Kids Institute, University of Western Australia, Perth.

**Keywords:** depression, anxiety, repeated measures, genetic epidemiology, molecular genetics

## Abstract

**Objective:**

To investigate the genetic architecture of internalizing symptoms in childhood and adolescence.

**Method:**

In 22 cohorts, multiple univariate genome-wide association studies (GWASs) were performed using repeated assessments of internalizing symptoms, in a total of 64,561 children and adolescents between 3 and 18 years of age. Results were aggregated in meta-analyses that accounted for sample overlap, first using all available data, and then using subsets of measurements grouped by rater, age, and instrument.

**Results:**

The meta-analysis of overall internalizing symptoms (INT_overall_) detected no genome-wide significant hits and showed low single nucleotide polymorphism (SNP) heritability (1.66%, 95% CI = 0.84–2.48%, n_effective_ = 132,260). Stratified analyses indicated rater-based heterogeneity in genetic effects, with self-reported internalizing symptoms showing the highest heritability (5.63%, 95% CI = 3.08%−8.18%). The contribution of additive genetic effects on internalizing symptoms appeared to be stable over age, with overlapping estimates of SNP heritability from early childhood to adolescence. Genetic correlations were observed with adult anxiety, depression, and the well-being spectrum (|*r_g_*| > 0.70), as well as with insomnia, loneliness, attention-deficit/hyperactivity disorder, autism, and childhood aggression (range |*r_g_*| = 0.42–0.60), whereas there were no robust associations with schizophrenia, bipolar disorder, obsessive-compulsive disorder, or anorexia nervosa.

**Conclusion:**

Genetic correlations indicate that childhood and adolescent internalizing symptoms share substantial genetic vulnerabilities with adult internalizing disorders and other childhood psychiatric traits, which could partially explain both the persistence of internalizing symptoms over time and the high comorbidity among childhood psychiatric traits. Reducing phenotypic heterogeneity in childhood samples will be key in paving the way to future GWAS success.

Internalizing disorders, including anxiety and depression, are substantial contributors to the global burden of disease.^1,2^ Although the estimated 12-month prevalence of depression and anxiety disorders in adults is 15%,^3^ internalizing disorders are also present in early life, with an estimated prevalence of 2% to 3% of depression and 6% to 7% of anxiety in childhood and adolescence.^4^ Prior to the diagnosis of internalizing disorders, as many as 1 in 5 children self report internalizing symptoms.^5^ These early symptoms of anxiety and depression appear to pose a long-term risk, as longitudinal studies show that internalizing symptoms in childhood are associated with mood disorders, anxiety, and suicidality in adulthood.^6–8^ Findings from twin research show that internalizing symptoms have a moderately strong genetic component. It is estimated that 40% to 50% of individual differences in internalizing symptoms are explained by genetic factors.^9–11^ Moreover, research suggests that both stability and change in anxious and depressive symptoms from early childhood to adulthood are genetically influenced.^10,12–14^ However, unlike adult anxiety and depression, investigation of the molecular genetic architecture of internalizing symptoms in early life has received little attention thus far, and, to date, only 2 studies have applied a genome-wide approach.^15,16^

Published in 2013 and 2014, the first genome-wide association studies (GWASs) on childhood internalizing symptoms did not identify any genome-wide significant hits for maternal-reported anxiety-related behaviors in children 7 years of age (N = 2,810)^15^ or internalizing problems in children 3 years of age (N = 4,596).^16^ Estimates of SNP-based heritability (the proportion of phenotypic variance captured by SNPs included in the GWAS), using genome-wide complex trait analysis (GCTA), were not robust in both studies.^15,16^ Other GCTA studies similarly show mostly inconsistent and broad estimates of SNP heritability, mainly due to small sample sizes.^17–22^ Large-scale GWASs have led to significant discoveries in adult samples, with now 102 variants identified for depression^23^ and 5 variants for anxiety.^24^ Given the comparable heritability estimates of adult and childhood internalizing phenotypes, the next step in this line of research is to increase childhood sample sizes in order to generate sufficient power to capture the small effects of common variants that have been observed in adult studies.

Here, we present a genome-wide association meta-analysis that aims to identify common genetic variants associated with the development and course of internalizing symptoms. The study combines repeated measurements of dimensional symptom scores from 22 independent cohorts of European ancestry, resulting in an overall sample of 64,641 individuals and 251,152 observations in children and adolescents between 3 and 18 years of age. All datasets were combined to produce a GWAS of overall internalizing symptoms (INT_overall_), with an effective sample size of 132,260. Stratified analyses were used to investigate age-, rater-, and instrument-specific genetic effects. The overall GWAS of INT_overall_ was followed up with gene-based analyses. Genetic overlap with external traits was examined by computing genetic correlations, with a focus on psychiatric phenotypes. Nonpsychiatric traits were also investigated if they were previously found to be genetically correlated with adult anxiety and depression.^23–25^ Finally, polygenic scores were computed to test prediction of internalizing symptoms in independent samples. With this study, we aim to gain insight into the genetic underpinnings of internalizing symptoms throughout childhood and adolescence to improve our understanding of the development and progression of internalizing disorders.

## METHOD

This project was preregistered at the Open Science Framework (https://osf.io/edas6). Minor deviations from the preregistration are explained in Supplement 1, available online.

### Sample and Univariate Analyses

The sample includes cohorts that are part of the EArly Genetics and Lifecourse Epidemiology (EAGLE) consortium behavior and cognition working group (https://www.eagle-consortium.org/)^26^ and additional cohorts with appropriate data. In total, 22 cohorts of European ancestry participated in the study. Ethical approval was provided by local committees at the cohort level. Many cohorts were longitudinal birth or childhood cohort studies with long-term follow-up and multiple raters, for example, mother, father, self, and teacher. Repeated assessments of internalizing symptoms within childhood and adolescence, from age 3 to age 18 years, were included. All cohorts performed univariate GWASs stratified by (1) age, (2) rater, and (3) instrument, with a minimum of 450 observations in each analysis. In the absence of diagnostic data, internalizing symptoms were dimensionally measured and positively scored on continuous scales, with higher scores indicating more internalizing symptoms. Data were not dichotomized into a case-control design, as this would have resulted in a reduction of statistical power.^27^ Detailed descriptions of the cohorts, phenotypic measures, and genotyping and imputation procedures can be found in Tables S1 to S6 and Supplement 1, available online.

Cohorts that included only unrelated subjects applied a linear regression model. Cohorts with a sample of related individuals corrected for nonindependence of observations by applying either a mixed linear model or a sandwich correction of the standard errors. Sex (ascertained through genotype) was included as a covariate in all univariate analyses. Further details about the univariate GWASs are provided in Supplement 1, and cohort-specific covariates are listed in Table S6, available online.

In total, 125 univariate GWASs were collated, with 251,152 observations based on 64,641 unique participants. The observations included ratings by mothers (40.7%), fathers (6.8%), teachers (18.3%), self (19.7%), and siblings (0.7%). An additional 13.8% of ratings were parental reports, for which the informant was either the mother or the father. Of the observations, 15.1% were in early childhood (3–6 years), 36.0% in mid childhood (7–10 years), 18.4% in late childhood (11–12 years), and 30.0% in adolescence (13–18 years). Twelve instruments were used to measure internalizing symptoms, of which the most commonly used were the Strengths and Difficulties Questionnaire (SDQ)^28^ (38.2%), Achenbach System of Empirically Based Assessment (ASEBA)^29^ (36.7%), and Rutter Children’s Questionnaires^30,31^ (8.2%).

### Meta-analyses and the Calculation of SNP Heritabilities Stratified by Age, Rater, and Instrument

Quality control for each univariate GWAS was performed using EasyQC (Supplement 1, available online).^32^ After quality control, most cohorts retained between 3.4 and 7.1 million autosomal SNPs per GWAS (Table S7, available online). An exception was the Philadelphia Neurodevelopmental Cohort, which retained fewer SNPs after merging data from different genotyping platforms. To account for dependency of repeated measurements of internalizing symptoms within cohorts, the N-weighed meta-analysis approach was applied.^33,34^ In short, two n × n matrices, representing sample overlap and phenotypic covariance within cohorts, were created, where n was the total number of univariate GWASs. As there was no overlap across cohorts, sample overlap and phenotypic covariance between cohorts were set to zero. Using the observed sample overlap within cohorts and their phenotypic covariance matrices, expected pairwise cross-trait intercept (CTI) values between GWASs were calculated. The pairwise CTI is approximately equal to the covariance between the test statistics from univariate GWASs. N-weighted meta-analyses were performed to obtain a multivariate test statistic per SNP, which represents a weighted sum of test statistics, adjusted by the CTI to account for sample overlap between GWASs. Formulas for the calculation of the multivariate test statistic for each SNP in the meta-analyses, the CTI between GWASs, and estimation of the effective sample size to account for repeated measurements (n_eff_) are provided in the Ip *et al*. supplementary text.^34^

A meta-analysis was performed based on the results of all available GWASs on internalizing symptoms: INT_overall_. SNP-based heritabilities (*h*^2^) were estimated using linkage disequilibrium score regression (LDSC),^35^ first for INT_overall_, and next based on results of meta-analyses stratified according to rater, age, rater-by-age, and instrument (TableS8, available online). To ensure that the stratified analyses had sufficient power, a sample size threshold was set so that the total number of observations (n_obs_) for each meta-analysis was at least 15,000. Rater-specific SNP heritabilities were estimated using assessments from parents (mother and/or father), mothers, fathers, teachers, and self, respectively. Age-specific SNP heritabilities focused on internalizing symptoms during early childhood (3–6 years), mid childhood (7–10 years), late childhood (11–12 years), and adolescence (13–18 years). Rater-by-age SNP heritabilities assessed age effects within and between raters, provided that the univariate N_obs_ exceeded 15,000. Finally, instrument-specific SNP heritabilities were calculated for SDQ, ASEBA, and Rutter for which the n_obs_ exceeded 15,000.

Genetic correlations across stratified GWAMAs were calculated using LDSC, but only if the *z* score of the heritability estimate was ≥4, given that the heritability z score is a good indicator of power and a score of <4 is considered too noisy for meaningful estimates.^36^

SNPs with minor allele frequency of <5% or n_eff_ of <15,000 were removed from further analyses.

### Gene-Based Analysis

Using summary statistics for INT_overall_, a MAGMA^37^ gene-based test (v1.8, implemented in FUMA^38^) was performed to identify genes with a significant effect on internalizing symptoms. The gene-based test applies a multiple regression model in which *p* values from individual SNPs in a gene are combined into a test statistic for each gene, while accounting for linkage disequilibrium between SNPs. European populations from the 1000 Genomes Phase III reference panel were used to estimate linkage disequilibrium. A total of 18,592 protein-coding genes were assessed for an association with internalizing symptoms. A Bonferroni correction was applied to correct for multiple testing (α = 0.05 / 18,592; *p* < 2.69×10^−06^).

### Tissue Expression and Gene-Set Analyses

Tissue enrichment and gene-set analyses were conducted in FUMA.^38^ The tissue enrichment analyses used 2 types of tissues from GTEx v8: namely, 30 general tissue types from multiple organs and 53 specific tissue types within these organs. A MAGMA gene-property test was performed to test one-sided relationships between cell type–specific gene expression and disease–gene associations. Bonferroni corrections were applied to correct for multiple testing for the general (α = 0.05 / 30; *p* < 1.7×10^−04^) and specific (α = 0.05 / 53; *p* < 9.4×10−^04^) tissue types.

The gene-set analysis was performed with default parameters in MAGMA v1.8. Gene-based *p* values were converted to *z* values, and a between-gene correlation matrix was used as input to perform gene-set enrichment tests. Predefined gene sets from the molecular signature database MsigDB v7.0 were used. In total, 15,484 gene sets were tested. A Bonferroni correction was applied to correct for multiple testing (α = 0.05 / 15,484; *p* < 3.210^−06^).

### Genetic Correlations With External Traits

Genetic correlations between internalizing symptoms and other phenotypes were investigated using publicly available summary statistics for a curated set of traits (N = 27). These included primarily adult psychiatric traits, in addition to other phenotypes selected based on previously identified correlations with adult anxiety and depression.^23–25^ In addition, we obtained summary statistics from the GWA meta-analyses of overall and rater-specific childhood and adolescent aggression,^34^ which were based on overlapping cohorts and similar statistical methods, and calculated genetic correlations with these traits. The external traits and source studies are summarized in Taable S9, available online. Summary statistics from INT_overall_ and INT_self_ (for which the z score of the *h*^2^ was ≥4^36^) were used. Genetic correlations were calculated using LDSC,^35^ which calculates genetic covariance between 2 traits based on all polygenic effects captured by included SNPs. Overlapping samples or population differences in GWAS summary statistics do not bias the computation of genetic correlations in LDSC. LDSC corrects for sample overlap by including a covariance matrix of the cross-trait LD score intercept, which is an estimate of sample overlap and phenotypic correlation. The genetic correlation estimate was based on the estimated slope from regressing the product of *z* scores from 2 GWASs on the LD score. The LD scores used were computed using 1000 Genomes Phase III European data.^36^ Genetic correlations were considered significant at *p* < 9.2610^−04^, after applying a Bonferroni correction for 54 independent tests.

### Sensitivity Analysis: Polygenic Score Prediction

Polygenic score prediction of INT_overall_ was tested as a sensitivity analysis. The Netherlands Twin Register (NTR) was used as the target sample to examine prediction of internalizing symptoms in childhood and adolescence. We considered maternal-reported internalizing symptoms at age 7 years (n = 3,845), and self-reported internalizing symptoms during adolescence (age 13–18 years, n = 2,679), using the ASEBA Child Behaviour Checklist and the Youth Self Report scales,^29^ respectively. A leave-one-cohort-out meta-analysis omitting data from NTR was performed for INT_overall_. The NTR target dataset was restricted to SNPs with minor allele frequency of >5% and imputation quality of *R*^2^ > 90%. Polygenic scores were constructed using LDpred,^39^ using a prior value of 0.5 to account for high polygenicity. Associations between polygenic scores of internalizing symptoms and internalizing problems were examined using generalized estimating equations as implemented in the “gee” package in R (v3.5.2). To account for relatedness in the target sample, the exchangeable working correlation matrix in gee was used, which applies a sandwich correction over the standard errors to account for clustering in the data. Age, sex, genotyping array, and the first 10 genetic principal components were included as covariates. Polygenic prediction was considered significant at *p* < .025, after applying a Bonferroni correction for 2 independent tests.

## RESULTS

### Overall Meta-analysis of Childhood and Adolescent Internalizing Symptoms

**FIGURE 1:**
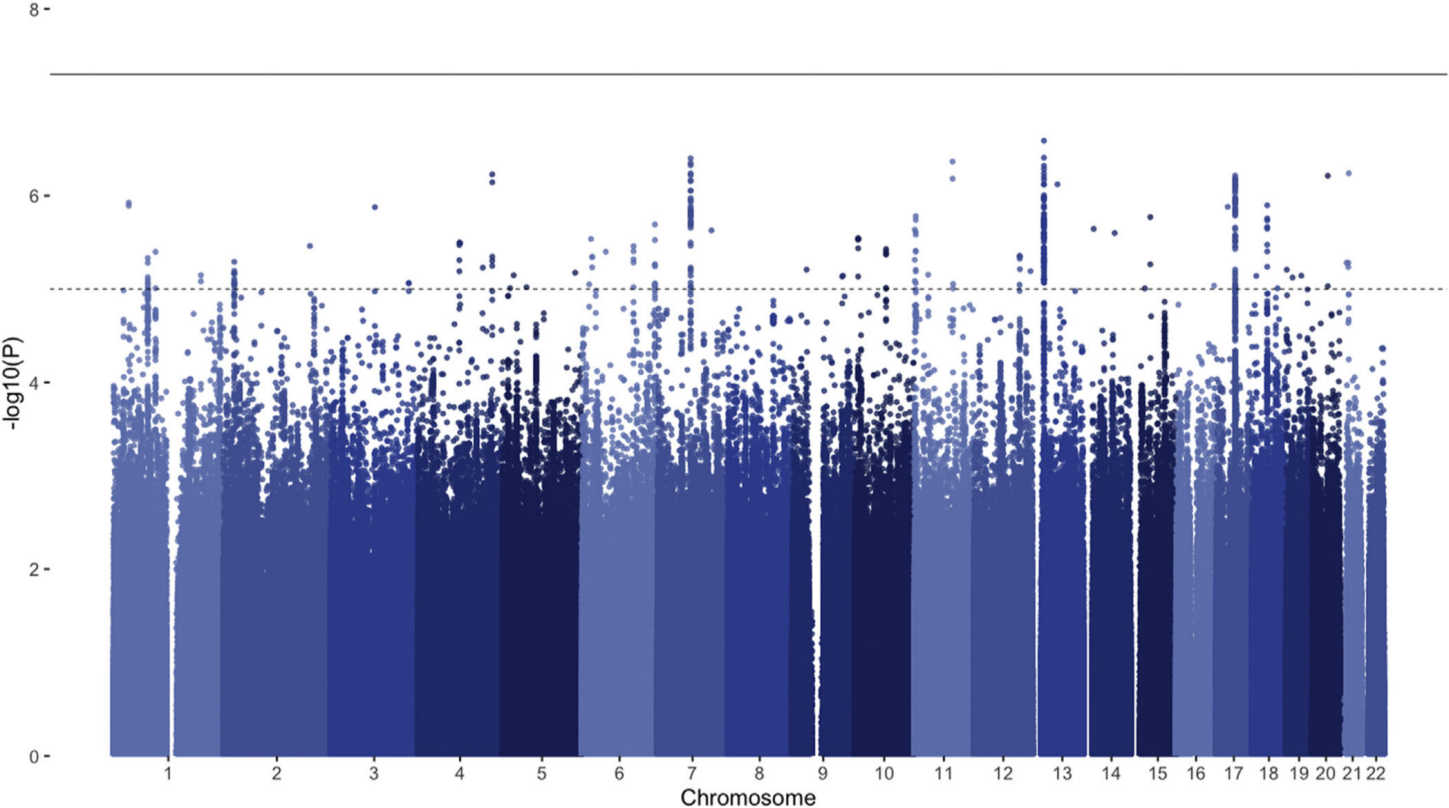
Manhattan Plot of the Meta-analysis of Overall Childhood and Adolescent Internalizing Symptoms (INT_overall_) **Note:** The solid line represents the significance threshold (p < 510^−08^); the dotted line represents the suggestive threshold (p < 110−^5^). Please note color figures are available online.

The genome-wide association meta-analysis of INT_overall_ found no genome-wide significant hits (Figure 1). Assuming a n_eff_ of 132,260, the SNP-based heritability of INT_overall_ was estimated at 1.66% (95% CI = 0.84%−2.48%). The mean χ^2^ statistic was 1.086, with an LDSC-intercept of 1.043 (standard error [SE] = 0.0075), indicating that a small part of the inflation in test statistics might have been due to confounding biases, such as population stratification.

### Stratified SNP Heritabilities and Within-Trait Genetic Correlations

**FIGURE 2:**
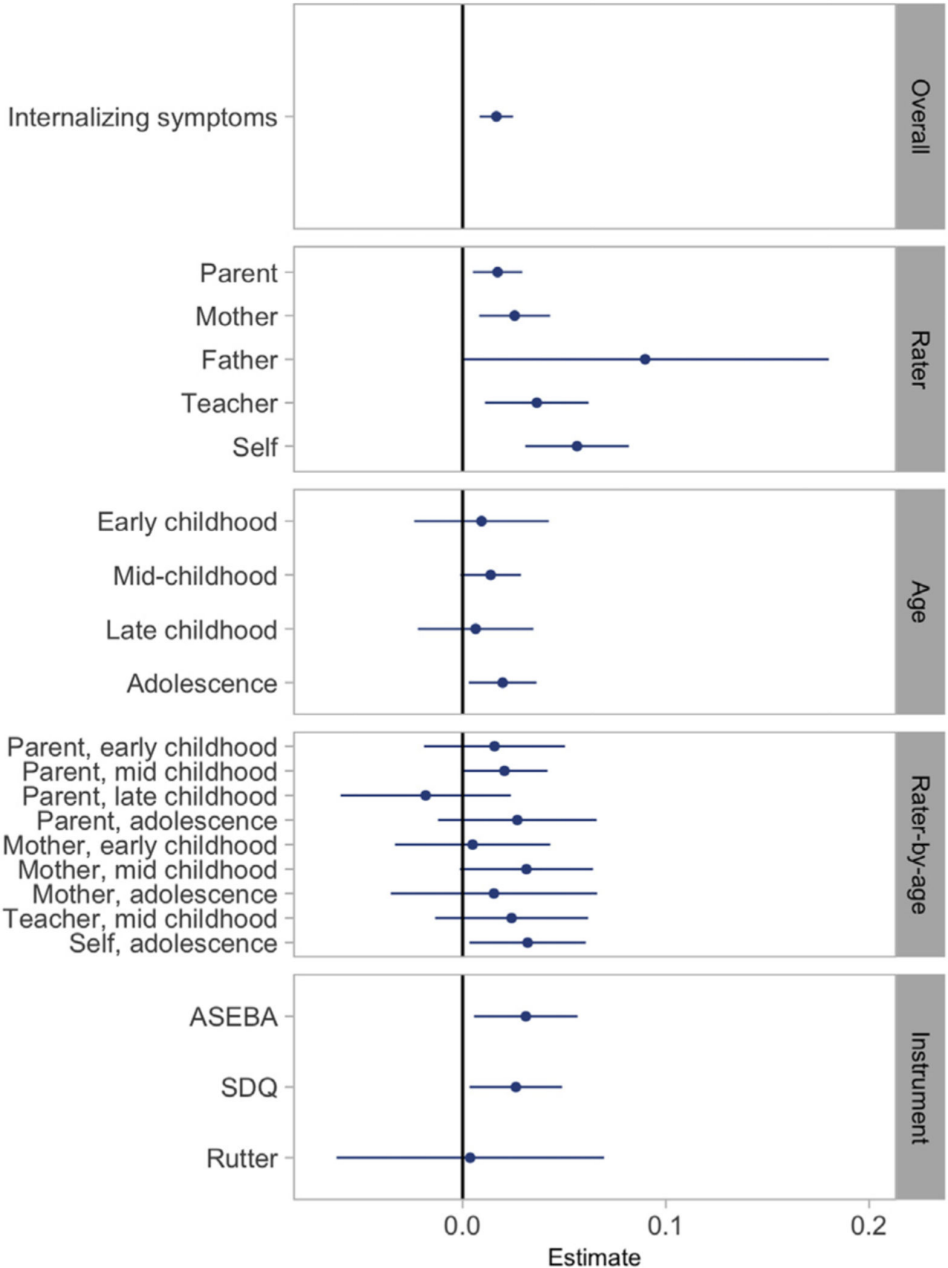
Single Nucleotide Polymorphism (SNP) Heritabilities Based on N-Weighted Meta-analyses of Internalizing Symptoms **Note:** Error bars represent 95% CI. ASEBA = Achenbach System of Empirically Based Assessment; SDQ = Strengths and Difficulties Questionnaire. Please note color figures are available online.

Estimates of SNP heritability from stratified meta-analyses are shown in Figure 2 and Table S8, available online. In rater-specific meta-analyses, self-reported internalizing symptoms showed the highest heritability (5.63%; 95% CI = 3.08%−8.18%), followed by teacher, maternal, and parental report, which were all significant. Although father-reported internalizing symptoms had the highest SNP heritability in rater-specific analyses (8.98%), the wide confidence intervals overlapped zero (0.06% to 18.02%). In age-specific meta-analyses, SNP *h*^2^ for internalizing symptoms in adolescence was highest (1.97%, 95% CI = 0.30%−3.64%), whereas estimates for early childhood, mid childhood, and late childhood were similar, but not robust to significance testing. In rater-by-age meta-analyses, self-reported internalizing symptoms during adolescence showed the highest SNP *h*^2^ (3.20%, 95% CI = 0.34%−6.06%). Instrument-specific meta-analyses showed that variance in internalizing symptoms explained by ASEBA and SDQ scales were comparable at ~3%. The estimate for Rutter was smaller (0.3%), but the difference was not substantial, based on the overlapping confidence intervals.

INT_overall_ and self-reported internalizing symptoms were highly genetically correlated (*r_g_* = 0.84, SE = 0.12, *p* = 2.08×10^−12^). The other stratified meta-analyses were insufficiently powered to estimate genetic correlations (heritability *z* score, <4).

### Gene-Based Analysis, Tissue Expression, and Gene-Set Analyses

The genome-wide gene-based analysis did not reveal any genes significantly associated with internalizing symptoms, but the top 10 genes are reported in Table S10, available online. MAGMA tissue expression analyses of 30 general and 53 specific tissue types did not show any statistically significant associations with internalizing symptoms (Table S11, available online). The gene-set analysis did not show any significant associations (Table S12, available online).

### Genetic Correlations With External Traits

**FIGURE 3:**
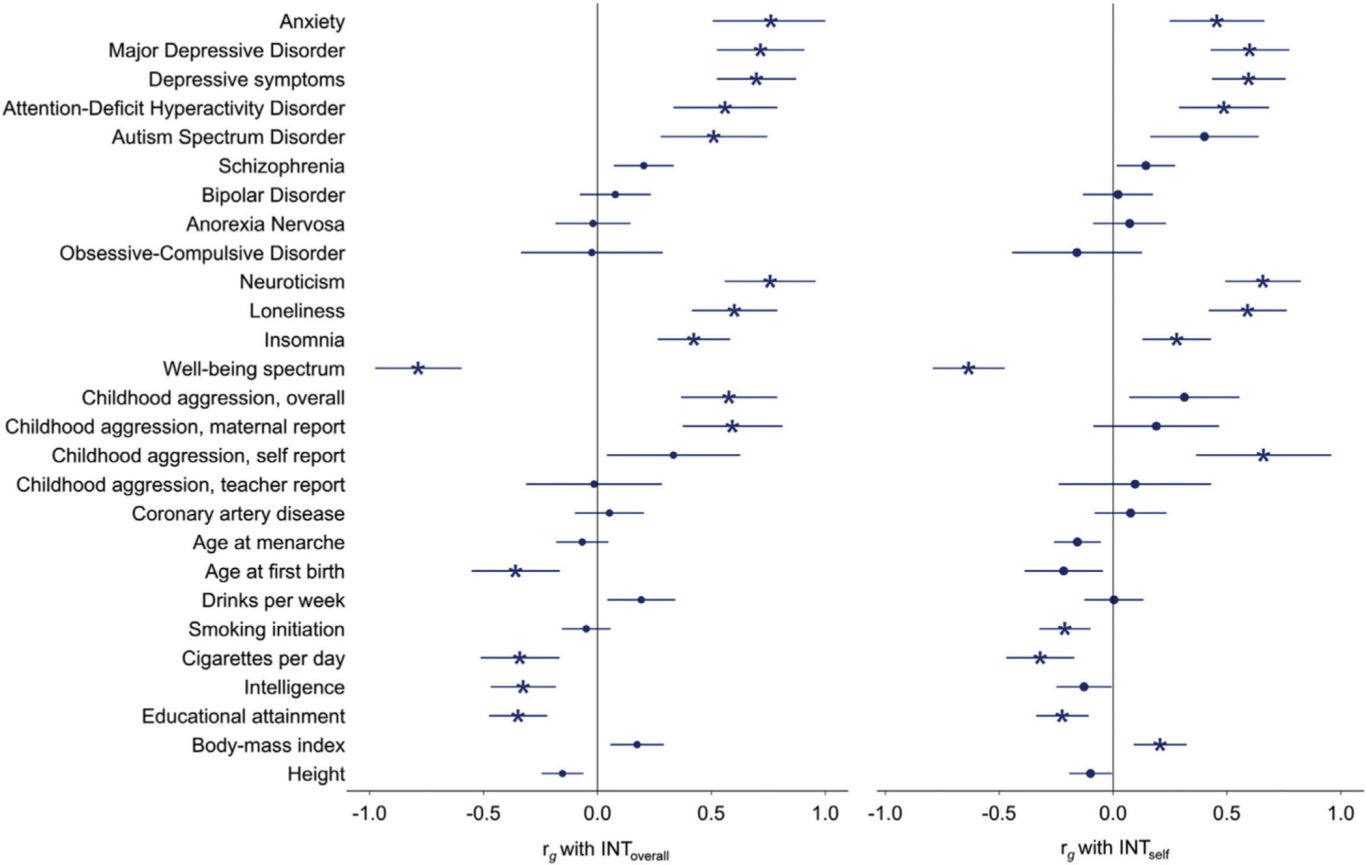
Genetic Correlations With External Phenotypes **Note:** The left panel shows genetic correlations with the meta-analysis for overall internalizing symptoms in childhood and adolescence (INT_overall_); the right panel shows genetic correlations with self-reported internalizing symptoms (INT_self_). Error bars represent 95% CIs. Correlations plotted with a star are statistically significant after correction for multiple testing. Please note color figures are available online.

Genetic correlations between INT_overall_ and INT_self_ (for which the *z* score of the *h*^2^ was ≥4^36^), and a set of preselected external traits are shown in Figure 3 and Table S13, available online. INT_overall_ held strong positive genetic correlations (*r_g_* > 0.7) with major depressive disorder, anxiety, and neuroticism, and a strong negative correlation (*r_g_* < −0.7) with the well-being spectrum. High correlations (|*r_g_*| > 0.5) with other adult and childhood psychiatric and psychological traits, including attention-deficit/hyperactivity disorder (ADHD), autism spectrum disorder (ASD), depressive symptoms, loneliness, and overall and maternal-reported aggression were found. Moderate genetic correlations (|*r_g_*| > 0.3) with insomnia, age at first birth, cigarettes per day, educational attainment, and intelligence were also observed. INT_self_ showed a similar, but generally weaker, pattern of genetic associations with external traits, with some exceptions. ASD, overall and maternal-reported aggression, age at first birth, and intelligence were correlated with INT_overall_ but showed weaker correlations with INT_self_, whereas self-reported aggression, smoking initiation, and body mass index (BMI) were correlated with INT_self_ but showed weaker or no correlation with INT_overall_.

### Polygenic Score Prediction

Prediction of internalizing symptoms in childhood and adolescence by polygenic scores based on INT_overall_ are shown in Table S14, available online. After correction for multiple testing, polygenic scores for INT_overall_ (N_eff_ = 132,260) were significantly associated with maternal-reported internalizing problems in children 7 years of age, and explained up to 0.38% of the phenotypic variance. Polygenic scores for INT_overall_ were not associated with self-reported internalizing problems in adolescence.

## DISCUSSION

This genome-wide association meta-analysis of childhood and adolescent internalizing symptoms included data from 64,641 children and adolescents between 3 and 18 years of age. The overall meta-analysis showed low SNP heritability (1.66%) and did not identify genome-wide significant loci or biological pathways for early life internalizing symptoms. Still, strong genetic correlations with external traits were observed, suggesting that childhood and adolescent internalizing symptoms share substantial genetic vulnerabilities with adult internalizing disorders and other childhood psychiatric traits, which could partially explain both the persistence of internalizing symptoms over time and the high comorbidity among childhood psychiatric traits. A more detailed look into the results of stratified analyses pointed to rater-based heterogeneous effects, indicating that in addition to further increases of sample size, approaches that reduce heterogeneity will be essential in future GWAS investigations.

The most striking findings of this study are the direction and strength of genetic correlations with external traits, which point to an overlapping genetic architecture between internalizing symptoms and other traits. The strong correlations may initially be surprising given the low SNP heritability observed here, but while SNP heritability estimates the overall variance in a trait explained by genome-wide SNPs, a genetic correlation reflects the extent to which the same set of genetic factors are involved in 2 traits. As such, even traits with low SNP heritability can have high genetic correlations if the underlying set of genetic factors influencing the traits are overlapping. Strong genetic correlations (|*r_g_*| > 0.7) with adult depression, anxiety, neuroticism, and the well-being spectrum were of note, and suggest a substantial shared genetic etiology between childhood internalizing symptoms and adult internalizing disorders and related traits, that has also been observed in previous studies.^40–42^ Viewed in combination with the overlapping estimates of SNP heritability from early childhood to adolescence in this study, these findings point to a stable set of genetic factors that partially explain the persistence of symptoms over time.

Comparisons with other adult psychiatric disorders showed high genetic correlations (|*r_g_*| > 0.5) with childhood-onset disorders ADHD and ASD, but no robust associations with bipolar disorder, obsessive-compulsive disorder, or anorexia nervosa. A small genetic correlation with schizophrenia was observed (*r_g_*= 0.2, *p* = .0025), which, albeit not significant due to the strict correction for multiple testing applied here, is in line with previous studies showing successful prediction of internalizing symptoms in childhood using polygenic scores for schizophrenia.^42–45^ The overall pattern of genetic correlations with other psychiatric traits is comparable to adult cross-disorder genetic correlations, where depression shows stronger associations with ADHD and ASD than with schizophrenia or bipolar disorder.^46^ It appears that, like adult depression, the broader (and perhaps also milder) symptomatology captured by dimensional measures of childhood internalizing symptoms shares fewer genetic similarities with severe and less common disorders such as schizophrenia, bipolar disorder, obsessive-compulsive disorder, and anorexia, but is more closely tied to childhood-onset disorders ADHD and ASD. This also resembles findings from the recent GWAS of total child psychiatric problems, which similarly found no robust genetic correlations with less common disorders.^47^ Correlations with other traits, including insomnia, loneliness, intelligence, educational attainment, cigarettes per day, and age at first birth were observed, as also seen in GWASs of adult depression and anxiety^23,24^; however, unlike adult depression, no robust associations with coronary artery disease, BMI, smoking initiation, or age at menarche were found. On the other hand, both BMI and smoking initiation held robust associations with INT_self_, for which ratings were available only during adolescence. This could indicate that genetic factors during adolescence are particularly important in these associations. Age-specific genetic effects may also explain why coronary artery disease was not associated with INT_overall_, in contrast to the small but robust genetic correlation that coronary artery disease shares with both adult depression and anxiety.^23,24^ Genetic innovation (the involvement of novel genetic variants) in adulthood may explain the genetic commonalities between adult internalizing disorders and coronary artery disease. Alternatively, the lack of genetic correlation between INT_overall_ and coronary artery disease, as well as age of menarche (which also genetically correlates with adult depression), could be due to a lack of power. This is indicated by the wide confidence intervals for some genetic correlations (Figure 3), which can be a consequence of low SNP heritability.

Focusing on childhood traits, as well as sharing high genetic correlations with childhood-onset disorders ADHD and ASD, internalizing symptoms were also highly correlated with childhood aggression. The high correlations observed across childhood traits indicate the presence of specific genetic effects that are common between childhood disorders within the neurodevelopmental spectrum. These shared genetic effects could partially explain the high comorbidity between psychiatric traits in childhood.^48–50^ In further examining the association between childhood internalizing symptoms and aggression, INT_overall_ shared high genetic correlations with overall and maternal-reported aggression, but not with teacher or self report. On the other hand, self-reported aggression and self-reported internalizing symptoms were highly correlated, whereas INT_self_ did not share robust associations with overall, teacher-reported, or maternal-reported aggression. These patterns of rater-stratified genetic correlations suggest that observed genetic effects on childhood phenotypes can vary substantially because of differences in the phenotype captured by different raters, with the same set of raters showing the highest correlation between traits.

The difficulty in identifying causal loci for early life internalizing symptoms is not novel, and resembles the trajectory of GWAS investigations of adult internalizing disorders. GWAS studies of adult depression also made slow progress because of limited sample sizes and heterogeneity.^51–53^ As depression has several potential sources of heterogeneity, including a diverse presentation of symptoms, large case-control sample sizes were required to achieve success in identifying specific genomic loci.^23,25^ GWAS studies of anxiety similarly saw increased success as sample sizes grew.^24,54^ Although the current study represents a substantial increase in sample size in comparison to previous GWASs of childhood internalizing phenotypes,^15,16^ the availability of childhood samples is still insufficient to lead to successful “brute force” GWASs such as those that are now available for adult internalizing disorders. Furthermore, in addition to heterogeneity due to a broad symptomatology, our findings indicate that GWASinvestigationsof childhood internalizingsymptoms are further disadvantaged by rater-based heterogeneous effects. Unlike adult studies in which measurements are typically self or clinician reports, childhood studies, particularly those focusing on early childhood, rely heavily on parent and teacher report, which act as an additional source of heterogeneity. Rater-based differences in genetic correlations with external traits have been discussed above. The current study also observed varying estimates of SNP heritability in rater-stratified analyses (Figure 2). Although these estimates did not appear to be significantly different (likely because of sample size limitations), the partial genetic correlation between INT_overall_ and INT_self_ points to incomplete overlap in relevant SNPs, indicating the presence of rater-specific genetic effects. In addition, polygenic scores based on INT_overall_ did not predict self-reported internalizing symptoms in the NTR cohort, which could also indicate heterogeneity between the target and discovery traits.^55^ Rater-specific genetic effects and rater disagreement on internalizing symptoms are noted in previous research,^56–59^ and rater-based heterogeneity is also reported in the GWAS of childhood aggression.^34^

Heterogeneous effects underlying childhood internalizing symptoms can be accounted for in multivariate GWAS approaches, but our study shows that current childhood samples seem unable to meet the power requirements of these types of analyses. Another way of reducing heterogeneity and helping signal detection is to focus on diagnoses. The case-control approach has proved to be more successful than dimensional measures in adult studies of depression and anxiety^23,24^ and overcomes the limitations of treating symptom scales as continuous traits. However, diagnostic data are currently not available for childhood phenotypes in large enough samples. Instead, we expect that reducing heterogeneity at phenotypic level will be key in paving the way to success in future GWAS investigations in childhood samples. This could be tackled by examining symptom-level phenotypes or separating childhood anxiety and depression into 2 distinct phenotypes. However, given the high genetic correlation between internalizing symptoms and both adult depression and anxiety, a more promising approach might be to jointly study childhood anxiety and depression, while eliminating heterogenous effects through factor analysis. Factor analysis can be used to derive a stable phenotype that captures the core behavior that multiple measurements (eg, from different informants or at different time points) have in common. This eliminates variability from rater, age, or situational effects. Evidence from both twin and molecular research shows that focusing on the common part of multiple assessments results in a more reliable phenotype that shows higher heritability than that captured by individual measurements separately.^40,58,60,61^ This way of managing rater bias has broader applicability in genetic studies within child psychiatry, but is dependent on the availability of multiple informants on behavior at one time point.

The findings of this study should be interpreted in light of several limitations. First, our multivariate GWAS approach relied on the assumption that meta-analyzing repeated measures of internalizing symptoms would increase power to detect genome-wide significant loci. This expectation was based on the reasonably strong correlations between measurements from different raters or at different ages. Instead, the burden of heterogeneity within childhood measurements amplified noise in the dataset. Combined with sample size limitations, this resulted in reduced statistical power, which is reflected in the low SNP heritability and lack of genome-wide significant findings. Second, the low estimate of SNP heritability in this study can also partly be explained by the methods: estimates of SNP heritability from summary statistics are typically lower than estimates from raw genotypic data, and potential overcorrection of biases in LDSC may have led to more conservative estimates. Third, the analyses in this study corrected for sex differences rather than investigating them through sex-differentiated analysis. We chose this approach because current evidence suggests that sex differences in genetic effects for psychiatric traits are either absent or small.^62^ However, sex-differentiated analyses in future work could provide insight into whether the influence of genetic factors on downstream biological processes or interplay between genetic risk and social environments can explain the different prevalence of internalizing behaviors in males and females. Fourth, due to the limited availability of diverse samples, the current findings are restricted to individuals of white European ancestry. An important goal for future GWASs is the funding and inclusion of multi-ancestry cohorts to allow better representation of diverse populations and to ensure broader applicability of findings.

To conclude, in this large GWAS of childhood and adolescent internalizing symptoms in population-based cohorts, no individual loci with strong associations with the outcome were detected. However, strong genetic correlations with adult internalizing traits and childhood psychiatric traits indicate that there is signal buried in the noise. Future GWAS success is likely to lie in reducing heterogeneity in childhood samples by focusing on a more stable phenotype of internalizing symptoms.

## Supplementary Material

Index

Supplement 1
