# Supplementary material for: Genome-wide Association Meta-analysis of Childhood and Adolescent Internalizing Symptoms
Source: J Am Acad Child Adolesc Psychiatry. Author manuscript; Available in PMC 2024 Feb 11. (PMC10859168; doi:10.1016/j.jaac.2021.11.035)
Supplement: Index [file NIHMS1913134-supplement-Index.pdf]

Index. Description of Supplementary Tables

Table	Description
S1	Cohorts included
S2	Maximum sample size contribution of each cohort
S3	Overview of instruments used
S4	Phenotypic descriptive statistics per GWAS
S5	Overview of genotyping, quality control and imputation
S6	Cohort-specific covariates
S7	Number of SNPs before and after EasyQC, per GWAS
S8	Stratified analyses of internalising symptoms
S9	List of external phenotypes for genetic correlations
S10	MAGMA gene-based test
S11	MAGMA tissue expression analyses
S12	MAGMA gene-set analysis
S13	Genetic correlations with external phenotypes
S14	Polygenic score analyses

Table S1 Cohorts included

Full name	Abbreviation	Country	Ascertainment
The 1958 Birth Cohort	1958BC	United Kingdom	population-based
Adolescent Brain Cognitive Development Study	ABCD	United States of America	population-based
The National Longitudinal Study of Adolescent to Adult Health	Add Health	United States of America	population-based
Avon Longitudinal Study of Parents and Children	ALSPAC	United Kingdom	population-based (family-based birth cohort)
Brain dEvelopment and Air polluTion ultrafine particles in scHool childrEn	BREATHE	Spain	population-based
Child and Adolescent Twin Study in Sweden	CATSS	Sweden	population-based (twin families)
Finnish Twin Cohort	FinnTwin12	Finland	population-based (twin families)
Generation R Study	Gen-R	the Netherlands	population-based
German Infant study on the influence of Nutrition Intervention PLUS environmental and genetic influences on allergy developpment / The influence of Life-style factors on the development of the Immune Ssytem and Allergies in East and West Germany	GINIplus/LISA	Germany	population-based (birth cohort)
Great Smoky Mountains Study	GSMS	United States of America	population-based
Institute for Behavioral Genetics	IBG	United States of America	population-based (twin families) and clinically ascertained
Norwegian Mother, Father and Child Cohort Study	MoBa	Norway	population-based (pregnancy cohort)
Michigan State University Twin Register	MSUTR	United States of America	population-based (twin families)
Mater University of Queensland Study of Pregnancy	MUSP	Australia	population-based (pregnancy cohort)
Northern Finland Birth Cohort 1986	NFBC1986	Finland	population-based (pregnancy cohort)
Netherlands Twin Register	NTR	the Netherlands	population-based (twin families)
Philadelphia Neurodevelopmental Cohort	PNC	United States of America	population-based
The Raine Study	Raine Study	Australia	population-based (pregnancy cohort)
Twin Early Development Study	TEDS	United Kingdom	population-based (twin families)
TRacking Adolescents' Individual Lives Survey	TRAILS	the Netherlands	population-based
Virginia Twin Study of Adolescent Behavioral Development	VTSABD	United States of America	population-based (twin families)
Young Finns Study	YFS	Finland	population-based (birth cohort)

Table S2 Maximum sample size contribution of each cohort

N_{unique} = number of unique individuals; N_{GWASs} = number of GWASs; N_{obs} = total number of observations

Cohort	N_{unique}	N_{GWASs}	N_{obs}
1958BC	4644	12	25684
ABCD	3677	2	3677
Add Health	4809	5	8213
ALSPAC	5707	9	42733
BREATHE	1156	1	1156
CATSS	7565	3	14712
FinnTwin12	1095	7	7033
Gen-R	2107	6	10075
GINIplus/LISA	1389	2	2583
GSMS	612	4	2190
IBG	777	4	2519
MoBa	6552	8	19514
MSUTR	1015	1	1015
MUSP	1225	2	2396
NFBC1986	3418	2	5838
NTR	4861	15	40723
PNC	3129	3	3129
Raine Study	1366	4	5407
TEDS	5579	18	34826
TRAILS	1660	8	11505
VTSABD	690	8	4616
YFS	1608	1	1608
Total	64641	125	251152

Table S3 Overview of instruments used

Instrument	Scale	Reference
Bristol Social Adjustment Guides (BSAG)	Depression / anxiety	Stott, D. H., & Sykes, E. G. (1963). Bristol Social-adjustment Guides. University of London Press.
Child and Adolescent Psychiatric Assessment (CAPA)	Depression	Angold, A., & Costello, E. J. (2000). The child and adolescent psychiatric assessment (CAPA). Journal of the American Academy of Child & Adolescent Psychiatry, 39(1), 39-48.
Achenbach System of Empirically Based Assessment (ASEBA): Child Behavior CheckList (CBCL), Teacher-Report Form (TRF), and Youth Self Report (YSR)	Internalising problems	Achenbach, T. M. (2009). The Achenbach system of empirically based assessment (ASEBA): Development, findings, theory, and applications. University of Vermont, Research Center for Children, Youth, & Families.
Center for Epidemiologic Studies Depression Scale (CES-D)	Depression	RADLOFF, L. (1997). Scale: A self-report depression scale for research in the general population. J Clin Exp Neuropsychol, 19, 340-356.
GOASSESS	Depression	Calkins, M. E., Merikangas, K. R., Moore, T. M., Burstein, M., Behr, M. A., Satterthwaite, T. D., ... & Qiu, H. (2015). The Philadelphia Neurodevelopmental Cohort: constructing a deep phenotyping collaborative. Journal of Child Psychology and Psychiatry, 56(12), 1356-1369.
Children's Manifest Anxiety Scale (MA)	Anxiety	Reynolds, C. R., & Richmond, B. O. (1979). Factor structure and construct validity of'What I think and feel': The revised children's manifest anxiety scale. Journal of Personality Assessment, 43(3), 281-283.
Multidimensional Peer Nomination Inventory (MPNI)	Depression	Pulkkinen, L., Kaprio, J., & Rose, R. J. (1999). Peers, teachers and parents as assessors of the behavioural and emotional problems of twins and their adjustment: the Multidimensional Peer Nomination Inventory. Twin Research and Human Genetics, 2(4), 274-285.
Rutter Children's Behaviour Questionnaires for parents and teachers (Rutter)	Internalising	Rutter, M. (1967). A children's behaviour questionnaire for completion by teachers: preliminary findings. Journal of child psychology and psychiatry, 8(1), 1-11.<br>Rutter M, Tizard J, Whitmore K (Eds) (1970). Education, health and behaviour. London: Longman
Screen for Child Anxiety Related Disorders (SCARED)	Anxiety	Birmaher, B., Khetarpal, S., Brent, D., Cully, M., Balach, L., Kaufman, J., et al. (1997). The<br>Screen for Child Anxiety Related Emotional Disorders (SCARED): Scale construction and<br>psychometric characteristics. Journal of the American Academy of Child and Adolescent Psychiatry<br>36: 545–553.
Strengths and Difficulties Questionnaire (SDQ)	Emotional problems	Goodman, R. (1997). The Strengths and Difficulties Questionnaire: a research note. Journal of child psychology and psychiatry, 38(5), 581-586.
Short Mood and Feelings Questionnaire (SMFQ)	Depression	Angold, A., Costello, E. J., Messer, S. C., Pickles, A., Winder, F., & Silver, D. (1995) The development of a short questionnaire for use in epidemiological studies of depression in children and adolescents. International Journal of Methods in Psychiatric Research, 5, 237 - 249.
Young Finns Study (YFS)	Internalising	Makkonen, T., Ruoppila, I., Rönkä, T., Timonen, S., Valvanne, L., & Österlund, K. (1981). Operation family. Mannerheim League of Child Welfare: Helsinki, Finland. Child Report A, 34.

Table S4 Phenotypic descriptive statistics per GWAS

Cohort	Rater	Age	N	% male	Age mean	SD	Median	IQR	Phenotype	Mean	Variance	Skewness	Kurtosis
1958BC - DIL	Mother	7	2156	48.47	7.334	0.242	7.253	7,168 - 7,420	Rutter	2.278	1.60727	0.5821971	-0.0352649
1958BC -WTCCC	Mother	7	2273	52.4	7.329	0.246	7.253	7,168 - 7,335	Rutter	2.243	1.58113	0.6570402	0.1030381
1958BC - DIL	Mother	11	2079	48.68	11.280	0.14	11.250	11,17 - 11,33	Rutter	3.065	2.20103	0.9519038	0.9328037
1958BC -WTCCC	Mother	11	2169	52.33	11.290	0.151	11.250	11,17 - 11,33	Rutter	3.065	2.20103	0.9519038	0.9328037
1958BC - DIL	Mother	16	1941	48.27	16.070	0.193	16.010	15,95 - 16,10	Rutter	1.854	1.74191	0.97631	0.6358359
1958BC -WTCCC	Mother	16	1973	51.85	16.070	0.193	16.010	15,95 - 17,20	Rutter	1.778	1.72953	1.12000	1.23880
1958BC - DIL	Teacher	7	2272	48.72	7.334	0.242	7.253	7,168 - 7,420	BSAG	3.341	4.32407	1.96765	4.48888
1958BC -WTCCC	Teacher	7	2375	51.66	7.329	0.246	7.253	7,168 - 7,335	BSAG	3.341	4.32407	1.96765	4.48888
1958BC - DIL	Teacher	11	2197	48.66	11.280	0.14	11.250	11,17 - 11,33	BSAG	3.259	4.03119	1.74003	3.26911
1958BC -WTCCC	Teacher	11	2256	52.48	11.290	0.151	11.250	11,17 - 11,33	BSAG	3.231	4.00591	1.78950	3.66498
1958BC - DIL	Teacher	16	1959	48.49	16.070	0.193	16.010	15,95 - 16,10	Rutter	1.078	1.43839	1.66122	2.88007
1958BC -WTCCC	Teacher	16	2041	51.79	16.070	0.193	16.010	15,95 - 17,20	Rutter	1.050	1.41610	1.67158	3.15934
ABCD	Parent	9	2392	47.69	9.679	0.4646224	9.750	9,250 - 10,083	CBCL	5.077	26.97091	1.780239	8.116743
ABCD	Parent	12	1285	45.08	11.030	0.440329	10.920	10,67 - 11,33	CBCL	5.542	34.11522	1.732664	6.649782
Add Health W1	Self	11 to 13	745	0.4053691	13.37248	0.3563188	13.40999	0.5804244	CES-D	4.4120810	12.89582	1.338859	2.569783
Add Health W1	Self	14 to 16	2545	0.4671906	15.56989	0.812891	15.581	1.338809	CES-D	5.6919450	18.08431	1.129507	1.528197
Add Health W2	Self	14 to 16	2055	0.448175	15.59840	0.8684523	15.66598	1.500342	CES-D	5.3493920	17.598410	1.180533	1.716146
Add Health W1	Self	17 to 18	1519	0.4858460	17.89815	0.5087915	17.83162	0.8323066	CES-D	5.8025020	18.0308	1.025044	1.018032
Add Health W2	Self	17 to 18	1349	0.4929577	17.88517	0.5418766	17.917	0.9144422	CES-D	5.779837	18.821670	1.060509	1.135166
ALSPAC	Mother	47 months	5,707	51.46	3.991	0.11635	4	4 - 4	SDQ	1.436	2.27571	1.25545	4.61077
ALSPAC	Mother	81m	5,300	51.09	81.405	1.30365	81	81 - 81	SDQ	1.477	2.67792	1.33225	4.73398
ALSPAC	Mother	97m	5,186	51.06	98.329	3.00712	97	97 - 99	SDQ	1.654	3.23261	1.21411	4.21231
ALSPAC	Mother	115m	5,284	50.45	115.716	1.4271	115	115 - 116	SDQ	1.436	2.83577	1.48438	5.38859
ALSPAC	Mother	140m	4,871	49.58	140.596	1.59266	140	140 - 141	SDQ	1.401	2.76106	1.50666	5.47593
ALSPAC	Mother	157m	4,779	49.68	157.898	2.20092	157	157 - 158	SDQ	1.371	2.71897	1.53001	5.49316
ALSPAC	Mother	198m	3,841	48.14	202.016	4.33833	200	198 - 205	SDQ	1.42	3.25363	1.6735	6.05967
ALSPAC	Teacher	7	3,537	50.55	99.953	3.77102	100	97 - 103	SDQ	1.312	3.55788	1.734	5.92329
ALSPAC	Teacher	10	4,228	50.26	133.927	3.94391	134	131 - 137	SDQ	1.235	3.29366	1.79473	6.28513
BREATHE	Parent	8	1156	52.59	8.01	0.57	7.990	7,52 - 8,52	SDQ	1.790	3.09000	1.173886	4.366369
CATSS	Mother	15	6272	47.86	15.470	0.532	15.000	15 - 16	SDQ	6.141	2.57100	1.81923	3.67632
CATSS	Father	15	1237	52.46	15.530	0.53	16.000	15-16	SDQ	6.075	2.20800	1.77246	3.61692
CATSS	Self	15	7729	46.91	15.470	0.52	15.000	15-16	SDQ	7.888	5.068	0.72636	-0.08182
FinnTwin12	Parent	12	1088	45.80	11.780	0.27355	11.790	11,58 - 12,02	MPNI	0.768	0.18600	0.742	3.46
FinnTwin12	Teacher	12	1095	45.40	11.550	0.30223	11.560	11,33 - 11,78	MPNI	0.698	0.25400	0.83600	3.50000
FinnTwin12	Teacher	14	851	44.90	14.230	0.20711	14.180	14,10 - 14,28	MPNI	0.610	0.25700	1.19700	4.54000
FinnTwin12	Self	14	990	45.90	14.200	0.14716	14.150	14,10 - 14,25	MPNI	0.722	0.14100	0.737	4.32
FinnTwin12	Self	17	1023	44.50	17.610	0.19643	17.610	17,45 - 17,74	MPNI	0.974	0.31000	0.726	3.46
FinnTwin12	Co-twin	14	978	46.40	14.200	0.15137	14.150	14,10 - 14,25	MPNI	0.661	0.16700	0.68800	3.42000
FinnTwin12	Co-twin	17	1008	44.70	17.610	0.19674	17.610	17,45 - 17,74	MPNI	0.826	0.25800	0.57200	3.16000
Gen-R	Mother	3	1822	50.55	3.041	0.09452612	3.008	3.060046-2.985813	CBCL	4.188	14.73114	1.917439	7.111043
Gen-R	Parent	6	2107	49.88	5.997	0.38016693	5.936	6.106346-5.776421	CBCL	5.253	29.01922	2.304842	8.514811
Gen-R	Mother	10	1792	50.95	9.706	0.2890987	9.659	9.812457-9.552361	CBCL	4.574	23.24786	1.971168	5.654143
Gen-R	Father	3	1626	50.12	3.051	0.10099251	3.022	3.071044-2.991312	CBCL	4.829	18.18892	1.467397	3.29202
Gen-R	Father	10	1459	51.47	9.787	0.31452324	9.747	9.883641-9.626283	CBCL	4.618	21.71183	1.867677	5.182892
Gen-R	Teacher	7	1269	47.60	6.662	1.30350916	6.149	7.612312-5.674695	TRF	2.671	16.45544	2.88016	12.16173
GINIplus/LISA	Parent	10	1389	51.84	10.03767	0.1986648	9.99	9.93-10.08	SDQ	1.835	3.699655	1.25335	4.335736
GINIplus/LISA	Self	15	1194	51.09%	15.21439	0.2993654	15.14	15.01-15.33	SDQ	2.12172	4.162479	1.062519	3.63407
GSMS	Parent	12	611	56.00	12.834	0.72652	12.972	12,118 - 13,246	CAPA	0.28922	0.46123	2.80814	8.63624
GSMS	Parent	15	572	56.00	15.496	0.57294	15.228	15,0719 - 15,9398	CAPA	0.234	0.43822	3.2948	11.22892
GSMS	Self	12	611	56.0	12.834	0.72652	12.972	12-12 - 13,25	CAPA	0.26471	0.42409	3.23964	13.28012
GSMS	Self	15	572	56.00	15.496	0.57294	15.228	15,07 - 15,94	CAPA	0.29144	0.46211	2.51145	6.00092
IBG	Parent	8	523	50.29	8.055	0.94827	8.000	7-9	CBCL	5.241	28.67748	1.65576	3.51103
IBG	Parent	11	566	52.47	11.018	0.85375	11.000	10-12	CBCL	5.189	27.95181	1.81947	4.33261
IBG	Parent	14	655	55.11	13.995	0.788862	14.000	13-15	CBCL	5.733	42.69456	1.99161	5.05307
IBG	Parent	16	777	60.62	16.338	0.62588021	16.000	15.5 - 16.5	CBCL	6.409	45.70342	1.49133	2.05593
MoBa	Mother	3	5840	51.84	3.056	0.06193275	3.033	3,019 - 3,085	CBCL	11.090	3.55883	1.202149	4.983507
MoBa	Mother	5	4518	52.22	5.252	0.2937818	5.083	5,000-5,417	CBCL	12.720	4.044756	1.847032	7.881518
MoBa	Mother	8	4573	52.2	8.131	0.12420899	8.083	8,083 - 8,167	SMFQ	14.730	5.72062	2.489333	12.25551
MoBa	Mother	8	4583	52.30	8.131	0.1241185	8.083	8,083-8,167	SCARED	5.980	1.40686	1.729183	7.699543
MSUTR	Mother	8	1015	50.25	7.850	0.89594	7.871	7.00000 - 8.81918	CBCL	5.459	27.59966	1.79008	4.244109
MUSP	Mother	5	1171	39.45	5.554	0.453	5.426	5,224-5,830	CBCL	3.627	8.11366	0.953477	3.61047
MUSP	Mother	14	1156	39.19	13.900	0.329	13.940	13,65 - 14,15	CBCL	3.607	8.05470	0.950038	3.57409
NFBC1986	Teacher	8	2420	48.64	NA	NA	NA	NA	Rutter	0.629	1.42157	2.366755	9.110403
NFBC1986	Self	16	3418	47.40	16.000	0.3719019	16.000	15.7 - 16.3	YSR	9.561	49.94220	1.111128	4.520208
NTR	Mother	3	5069	47.49	3.350	0.27834	3.296	3,135-3,507	CBCL	7.991	39.04648	1.19362	4.77547
NTR	Mother	7	4526	47.55	7.444	0.39431	7.365	7,176-7,666	CBCL	4.658	23.43077	1.99518	8.98136
NTR	Mother	10	4246	47.27	9.897	0.37040	9.927	9,725-10,144	CBCL	4.901	29.15657	2.00311	8.27543
NTR	Mother	12	3651	47.38	12.000	0.51333	12.010	11,82-12,31	CBCL	4.348	25.10763	2.02469	8.24193
NTR	Father	3	3713	47.40	3.389	0.27975	3.335	3,176-3,548	CBCL	7.468	36.72428	1.29937	5.27014
NTR	Father	7	3735	46.88	7.446	0.39724	7.368	7,176-7,655	CBCL	3.573	15.95115	2.24832	10.69862
NTR	Father	10	3471	46.66	9.903	0.37396	9.936	9,730-10,155	CBCL	3.667	19.15014	2.34145	10.97536
NTR	Father	12	2978	46.31	12.000	0.50935	12.010	11,82-12,30	CBCL	3.410	19.36868	2.53214	12.27869
NTR	Teacher	7	1993	49.72	7.284	0.32203	7.302	7,083-7,477	TRF	3.839	17.84905	1.76685	7.08285
NTR	Teacher	10	2326	46.99	9.675	0.49958	9.771	9,300-10,048	TRF	4.393	24.09680	1.79059	7.03989
NTR	Teacher	12	2437	46.78	12.020	0.44190	12.080	11,87-12,28	TRF	3.971	22.62107	2.17336	9.30039
NTR	Self	12	1206	48.09	12.200	0.42255	12.250	12,00-12,47	YSR	8.498	46.70000	1.19720	4.77875
NTR	Self	14	1287	43.43	14.370	0.47265	14.440	14,18-14,73	YSR	8.560	45.92619	1.25918	5.05008
NTR	Self	16	1479	44.62	16.480	0.45441	16.600	16,31-16,80	YSR	8.857	50.78999	1.41196	5.89004
NTR	Self	18	1455	40.07	17.820	0.55440	17.840	17,29-18,26	YSR	9.254	52.81941	1.23214	4.87133
PNC	Parent	8 to 10	1059	0.5741265	8.924	0.832899	9.000	8-10	GOASSESS	0.3267233	0.7494837	2.942433	8.195316
PNC	Self	11 to 13	966	0.515528	12.04141	0.8004815	12.000	11-13	GOASSESS	0.7546584	1.341818	1.473427	1.094241
PNC	Self	14 to 16	1104	0.4773551	15.03804	0.804792	15.000	14-16	GOASSESS	1.112319	1.961988	0.9133983	-0.6024446
Raine Study	Parent	5	1363	51.36	5.900	0.19015	5.890	5.820-5.980	CBCL	5.886	29.91053	1.46245	5.29886
Raine Study	Parent	8	1347	51.22	8.080	0.34893	8.090	7.800-8.360	CBCL	6.260	35.55502	1.5916	6.16177
Raine Study	Parent	10	1366	51.90	10.580	0.19897	10.530	10.47-10.63	CBCL	5.822	35.01815	1.7216	6.57166
Raine Study	Self	13	1331	51.31	14.040	0.20911	14.060	13.99-14.14	YSR	5.413	36.95250	2.13076	9.24565

TEDS	Parent	7	5579	47.89	7.060	0.25059	7.04	5.57 - 8.62	SDQ	1.81657	3.29992	1.0714	1.16339
TEDS	Parent	9	2673	46.19	9.020	0.28927	9	8.08 - 11.34	SDQ	1.693	3.30000	1.345812	1.638
TEDS	Parent	12	4644	47.15	11.28	7.02E-01	11.440	9.79 - 15.62	SDQ	1.776	3.65000	1.311	1.616
TEDS	Teacher	7	4586	48.36	7.20026	2.76E-01	7.22793	6.57 - 8.03	SDQ	1.302	3.15700	1.688521	2.954878
TEDS	Teacher	9	2224	46.33	9.0334	2.88E-01	9.01437	8.46 - 10.14	SDQ	1.313	3.35800	1.705	2.837
TEDS	Teacher	12	3836	46.63	11.53216	6.62E-01	11.6386	10.12 - 14.09	SDQ	1.190	3.15200	1.921	3.924
TEDS	Self	9	2633	53.80	9.01695	2.89E-01	9.00205	8.08 - 11.34	SDQ	3.223	5.62560	0.607126	-0.260724
TEDS	Self	12	4631	53.14	11.2758	6.98E-01	11.436	9.79 - 15.62	SDQ	2.1903	4.35762	1.020176	0.634684
TEDS	Self	16	3999	55.82	16.32395	6.80E-01	16.47912	14.91 - 18.76	SDQ	2.779	5.10625	0.737382	-0.037459
TRAILS	Mother	11	1532	51.70	11.079	0.53669	11.003	10.671 - 11.418	CBCL	9.059	50.01353	1.16473	4.38332
TRAILS	Mother	13	1411	50.67	13.356	0.60722	13.383	12.945 - 13.789	CBCL	7.318	41.93058	1.33158	5.11779
TRAILS	Mother	16	1324	51.59	16.113	0.65172	16.048	15.655 - 16.469	CBCL	7.031	46.41703	1.43196	5.15202
TRAILS	Teacher	11	1487	51.31	11.050	0.52553	10.962	10.667 - 11.369	TRF	11.909	160.09741	1.08153	3.73831
TRAILS	Teacher	13	1282	52.42	13.317	0.61234	13.329	12.893 - 13.762	TRF	14.460	190.46251	1.08639	3.99189
TRAILS	Teacher	16	1017	53.20	15.991	0.5836	15.959	15.592 - 16.337	TRF	15.479	186.42500	1.00975	3.9256
TRAILS	Self	11	1663	51.89	11.088	0.54219	11.006	10.68 - 11.443	YSR	11.769	53.78058	0.72384	3.11163
TRAILS	Self	13	1661	51.90	13.373	0.60644	13.385	12.975 - 13.818	YSR	10.645	56.31695	0.96509	4.00263
TRAILS	Self	16	1610	51.49	16.138	0.66922	16.055	15.671 - 16.492	YSR	9.839	59.55625	1.09756	4.27731
VTSABD	Parent	13	549	40.53	13.911	0.73339	14.081	12,827 - 14,995	CAPA	1.180	1.67363	1.01644	0.26936
VTSABD	Parent	13	552	40.53	13.911	0.73339	14.081	12,827 - 14,995	Rutter	2.246	3.91378	0.9917718	0.65124435
VTSABD	Parent	16	569	42.60	16.088	0.5376	16.194	15,318 - 17,071	CAPA	1.218	1.90665	1.08184	0.3586938
VTSABD	Parent	16	590	42.60	16.088	0.5376	16.194	15,318 - 17,071	Rutter	2.020	3.98261	1.03160	0.63672412
VTSABD	Self	13	547	40.53	13.911	0.73339	14.081	12,827 - 14,995	CAPA	0.985	1.47231	1.29986	1.13579
VTSABD	Self	13	553	40.53	13.911	0.73339	14.081	12,827 - 14,995	MA	2.022	3.03214	0.8736504	0.34390084
VTSABD	Self	16	579	42.60	16.088	0.5376	16.194	15,318 - 17,071	CAPA	1.425	2.10637	0.80402	-0.3716444
VTSABD	Self	16	577	42.60	16.088	0.5376	16.194	15,318 - 17,071	MA	2.005	3.11629	0.89654	0.46710943
YFS	Mother	6 to 21	1608	45.4	13.53731	5.03786	15	9-18	YFS	0.00317	0.93103	0.22778	16.51297

Cohort= DIL: Diabetes and Inflammation Laboratory, WTCCC: Welcome Trust Case Control Consortium, W1: Wave 1, W2: Wave 2

Phenotype= BSAG: Bristol Social Adjustment Guides, CAPA: Child and Adolescent Psychiatric Assessment,CBCL: Child Behavior Checklist, CES-D: Center for Epidemiological Studies Depression Scale, MA: Children's Manifest Anxiety Scale, MPNI: Multidimensional Peer Nomination Inventory, SDQ: Strengths and Difficulties Questionnaire, SCARED: Screen for Anxiety Related Disorders, SMFQ: Short Mood and Feelings Questionnaire, TRF: Teacher Report Form, YFS: Young Finns Study, YSR: Youth Self Report

Table S5 Overview of genotyping, quality control, and imputation
MAF = minor allele frequency; HWE = Hardy-Weinberg Equilibrium; SD = standard deviations

Cohort	Array(s)	Pre-imputation quality control							Imputation	
		Variant filters				Sample filters				
		Call rate	MAF	HWE P-value	Other	Call rate	Heterozygosity	Other	Reference panel	Software
1958BC	Illumina HumanHap 550<br>Illumina Human 1M<br>Affymetrix 6.0	95%	0.01	1E-06		95%	+/- 4 SD from mean	relatedness, failed sex check	1000 Genomes Phase 1 Version 3, global	IMPUTE v2
ABCD	Affymetrix NIDA SmokeScreen Array	95%	0.01	1E-06		95%	0,45 < F < 0,45	cryptic relatedness (pi > 0,2), failed sex check	1000 Genomes Phase 3 Version 5, European	Minimac 4
Add Health	Illumina HumanOmni1<br>Illumina HumanOmni2.5	98%	0.05	1E-06		95%	median + 3*IQR	gender mismatch, duplicates, relatedness	1000 Genomes Phase 3 Version 5, global	Minimac3 / MACH
ALSPAC	Illumina Human Hap 550-quad	99%	0.01	5E-07		97%	0,0375 < F < 0,27	relatedness, duplicates	1000 Genomes Phase 1 Version 3, global	IMPUTE v2
BREATHE	Illumina HumanCore BeadChip WG-330-1101	95%	0.01	1E-06	LRR SD < 0,3	97%	+/- 4 SD from mean	relatedness	1000 Genomes Phase 1 Version 3, European	IMPUTE v2
CATSS	Illumina PsychArray<br>- Infinium Core-24 BeadChip (265,000 markers)<br>- Infinium Exome-24 BeadChip (245,000 markers)<br>- 50,000 markers associated with common psychiatric disorders	98%	0.01	1E-06	>10% discordant genotypes among cross-batch duplicate samples; discordant genotype among MZ twins; absolute allele frequency difference >10% compared to 1000G EUR	98%	-0,2 < F < 0,2	+/- 6 SD from mean of average sample relatedness	1000 Genomes Phase 3 Version 5, European	Minimac3 / MACH
FinnTwin12	CCC: Illumina Human670-QuadCustom v1 A<br>FTALCO: Illumina Human670-QuadCustom v1 A<br>FT: Illumina HumanCoreExome-12 v1.1 A<br>gftmp: Illumina HumanCoreExome-12 v1.0 A<br>KAP: Illumina HumanCoreExome-24 v1.0 A	98%	0.01	1E-06		97%	+/- 3 SD from mean; +/- 4 SD from mean; -0,03 < F < 0,05		1000 Genomes Phase 3 Version 5, global	Minimac3 / MACH
Gen-R	Illumina 610K Quad Chip (cord blood at birth)<br>Illumina 660k Quad Chip (venous blood at follow-up collection)	95%	0.01	1E-07		97.5%	+/- 4 SD from mean	relatedness, duplicates	1000 Genomes Phase 3 Version 5, European	MACH
GINIplus/LISA	Affymetrix 5.0<br>Affymetrix 6.0	95%	0.01	1E-05		95%	+/- 4 SD from mean		1000 Genomes Phase 3 Version 5, European	IMPUTE v2
GSMS	Illumina Human660W-Quad v1	95%	0.01	1E-08	strand ambiguity	90%	+/- 5 SD from mean	relatedness	1000 Genomes Phase 3 Version 5, European	IMPUTE v2
IBG	Axiom Precision Medicine Research Array<br>Affymetrix Genome-Wide Human SNP Array 6.0	95%	0.01	NA		99%	NA		1000 Genomes Phase 3 Version 5, European	Minimac3 / MACH
MoBa	Illumina HumanCoreExome-12<br>Illumina HumanCoreExome-24	98%	0.01	1E-07		98%	+/- 4 SD from mean	cryptic relatedness (pi > 0.1), sex mismatch	1000 Genomes Phase 3 Version 5, global	IMPUTE v2
MSUTR	Illumina HumanCoreExome	99%	0.05	1E-06		95%	-0,2 < F < 0,2		1000 Genomes Phase 3 Version 5, European	Minimac3 / MACH
MUSP	PsychChip v1.0	95%	0.01	1E-06	strand ambiguity	95%	Y	relatedness (pi > 0,2)	1000 Genomes Phase 3 Version 5, European	Minimac3 / MACH
NFBC1986	Illumina HumanOmniExpressExome-8v1.2	99%	NA	0.0001		95%	Y	relatedness (pi > 0,2)	1000 Genomes Phase 3 Version 5, European	IMPUTE
NTR	Perlegen Affymetrix<br>Affymetrix 6.0<br>Affymetrix Axiom<br>Illumina 660<br>Illumina 1M<br>Illumina GSA<br>GoNL sequence	95%	0.01	1E-05	strand ambiguity; number of Mendelian errors > 20; +/- 3 SD from mean Mendelian error rate	90%	-0,1 < F < 0,1		1000 Genomes Phase 3 Version 5, European	Minimac3 / MACH
PNC	Human1M-Duov3_B<br>HumanOmniExpressExome-8v1_A<br>HumanOmniExpress-12v1_A<br>HumanOmniExpress-12v1-1_B<br>Human610-Quadv1_B<br>BDCHP-1X10-HUMANHAP550<br>HumanHap550v3_A	98%	NA	NA		98%	NA	failed sex check, relatedness	1000 Genomes Phase 3 Version 5	Minimac3 / MACH
Raine Study	Illumina Human 660W Quad Array	95%	0.01	1E-06	strand ambiguity	95%	h>0,32	cryptic relatedness (pi ≥ 0,1875)	1000 Genomes Phase 3 Version 5, European	Minimac3 / MACH
TEDS	Illumina HumanOmniExpressExome-8v1.2<br>AffymetrixGeneChip 6.0 SNP	98%	0.01	1E-05		98%	-0,1 < F < 0,1	relatedness (KING kinship coefficient < 0,0884 [r~=0,25])	1000 Genomes Phase 3 Version 5, European	MACH
TRAILS	Illumina Cyto SNP12 v2 + TaqMan	95%	0.01	0.0001		95%	+/- 4 SD from mean	relatedness (pi > 0,35)	1000 Genomes Phase 1 Version 3, European	IMPUTE v2
VTSABD	Illumina Human660W-Quad v1	95%	0.01	1E-08	strand ambiguity	90%	+/- 5 SD from mean	relatedness	1000 Genomes Phase 3 Version 5, European	IMPUTE v2
YFS	Illumina BeadChip Human670K	95%	0.01	1E-06		95%	Y	relatedness (pi > 0,2)	1000 Genomes Phase 1 Version 3, global	IMPUTE

Table S6 Cohort-specific covariates

PCs = principal components; GRM = genetic relatedness matrix

Cohort	Covariates
1958BC	sex, site, first five ancestry-based PCs
ABCD	sex, age, batch, first five ancestry-based PCs
Add Health	sex, age, first five ancestry-based PCs
ALSPAC	sex, age, first five ancestry-based PCs
BREATHE	sex, age, first five ancestry-based PCs
CATSS	sex, first five ancestry-based PCs
FinnTwin12	sex, age, kinship matrix
Gen-R	sex, age, first five ancestry-based PCs
GINIplus/LISA	sex, age, study
GSMS	age, sex, first five ancestry-based PCs
IBG	age, sex, site, first five ancestry-based PCs
MoBa	sex, age, batch (when required), first five ancestry-based PCs
MSUTR	sex, age, first five ancestry-based PCs, GRM
MUSP	sex, age, first ten ancestry-based PCs
NFBC1986	sex, age, first four MDS dimensions
NTR	age, sex, genotyping array, first five ancestry-based PCs, 2 GRMs
PNC	sex, age, first five ancestry-based PCs
Raine Study	sex, age, first five ancestry-based PCs
TEDS	sex, age, first five ancestry-based PCs
TRAILS	sex, age, first five ancestry-based PCs
VTSABD	sex, age, first five ancestry-based PCs, GRMs
YFS	sex, age, first five ancestry-based PCs

Table S7 Number of SNPs before and after EasyQC, per GWAS

Cohort	Rater	Mean age (rounded)	Sub-analysis	Number of SNPs before QC	after QC
1958BC	Teacher	7	Study 1	38045094	4952213
1958BC	Teacher	11	Study 1	38045094	4952213
1958BC	Teacher	16	Study 1	38045094	4421770
1958BC	Mother	7	Study 1	38045094	4952213
1958BC	Mother	11	Study 1	38045094	4926068
1958BC	Mother	16	Study 1	38045094	4421770
1958BC	Teacher	7	Study 2	38047659	5396498
1958BC	Teacher	11	Study 2	38047659	5396498
1958BC	Teacher	16	Study 2	38047659	5326787
1958BC	Mother	7	Study 2	38047659	5396498
1958BC	Mother	11	Study 2	38047659	5396498
1958BC	Mother	16	Study 2	38047659	4812206
ABCD	Parent	9		47098604	4648812
ABCD	Parent	12		47098604	4137718
Add Health	Self	12	Wave 1	42536462	3736021
Add Health	Self	15	Wave 1	42965354	5657465
Add Health	Self	17	Wave 1	42833772	4894482
Add Health	Self	15	Wave 2	42917514	5657465
Add Health	Self	17	Wave 2	42792834	4894482
ALSPAC	Teacher	7		27178589	5831802
ALSPAC	Teacher	10		27178600	5833981
ALSPAC	Mother	115m		27178649	7136691
ALSPAC	Mother	140m		27178634	5833291
ALSPAC	Mother	157m		27178619	5833455
ALSPAC	Mother	198m		27178596	5832509
ALSPAC	Mother	47m		27178676	7137279
ALSPAC	Mother	81m		27178657	7137189
ALSPAC	Mother	97m		27178656	7136804
BREATHE	Parent	8		37759387	5093532
CATSS	Father	15		12938273	5140498
CATSS	Mother	15		12938273	6909050
CATSS	Self	15		12938273	6911168
FinnTwin12	Co-twin	14		47075775	4210866
FinnTwin12	Co-twin	17		47075775	5293702
FinnTwin12	Parent	12		47075775	5294080
FinnTwin12	Self	14		47075775	4210574
FinnTwin12	Self	17		47075775	5294345
FinnTwin12	Teacher	12		47075775	5293238
FinnTwin12	Teacher	14		47075775	4211117
Gen-R	Teacher	7		34349995	4142231
Gen-R	Father	10		28856182	5032040
Gen-R	Mother	10		28856182	5031920
Gen-R	Father	3		28856182	5031971
Gen-R	Mother	3		28856182	5031898
GINIplus/LISA	Parent	10		30056331	5025912
GINIplus/LISA	Self	15		30056331	5025047
GSMS	Self	13		6838237	3685008
GSMS	Self	16		6748337	3645420
GSMS	Parent	13		6310658	3401135
GSMS	Parent	13		6748337	3645420
IBG	Parent	8		8200256	3986519
IBG	Parent	11		8199955	3695992
IBG	Parent	14		8198247	3745317
IBG	Parent	16		8888183	3418656
MoBa	Mother	3	Chip 1	39131578	6022644
MoBa	Mother	5	Chip 1	39131578	6025308
MoBa	Mother	8	Chip 1, SMFQ*	39131578	6025498
MoBa	Mother	8	Chip 1, SCARED*	39131578	6025367
MoBa	Mother	3	Chip 2	39131578	6028506
MoBa	Mother	5	Chip 2	39131578	5333152
MoBa	Mother	8	Chip 2, SMFQ*	39131578	5329251

MoBa	Mother	8	Chip 2, SCARED*	39131578	5329051
MSUTR	Mother	8		18529974	5013388
MUSP	Mother	5		47099760	4918217
MUSP	Mother	14		47099760	4917032
NFBC1986	Self	16		12796984	6177075
NFBC1986	Teacher	8		12402368	6177066
NTR	Mother	3		4934427	4594974
NTR	Mother	7		4933175	4593844
NTR	Mother	10		4935567	4595911
NTR	Mother	12		4935518	4595850
NTR	Father	2		4933964	4594618
NTR	Father	7		4933103	4593685
NTR	Father	10		4934823	4595272
NTR	Father	12		4936227	4596581
NTR	Teacher	7		4423617	4112565
NTR	Teacher	10		4935834	4596230
NTR	Teacher	12		4936782	4597092
NTR	Self	12		4433962	4122188
NTR	Self	14		4427483	4116135
NTR	Self	16		4422899	4111933
NTR	Self	18		4427575	4116154
PNC	Parent	9		42963164	2557981
PNC	Self	12		42961641	1849605
PNC	Self	15		42964071	2557981
Raine Study	Parent	10		48096953	5168223
Raine Study	Self	13		48096953	5168223
Raine Study	Parent	5		48096953	5168223
Raine Study	Parent	8		48096953	5168223
TEDS	Parent	7	Chip 1	9220773	5562040
TEDS	Parent	9	Chip 1	9220773	4957940
TEDS	Parent	12	Chip 1	9220773	4957940
TEDS	Teacher	7	Chip 1	9220773	4957940
TEDS	Teacher	9	Chip 1	9220773	3968868
TEDS	Teacher	12	Chip 1	9220773	4957940
TEDS	Self	9	Chip 1	9220773	4957940
TEDS	Self	12	Chip 1	9220773	4957940
TEDS	Self	16	Chip 1	9220773	4957940
TEDS	Parent	7	Chip 2	9264517	5663362
TEDS	Parent	9	Chip 2	9264517	5035667
TEDS	Parent	12	Chip 2	9264517	5663362
TEDS	Teacher	7	Chip 2	9264517	5663362
TEDS	Teacher	9	Chip 2	9264517	5035667
TEDS	Teacher	12	Chip 2	9264517	5663362
TEDS	Self	9	Chip 2	9264517	5035667
TEDS	Self	12	Chip 2	9264517	5663362
TEDS	Self	16	Chip 2	9264517	5663362
TRAILS	Self	11		47100222	5024277
TRAILS	Self	13		47100222	5023520
TRAILS	Self	16		47100222	5022025
TRAILS	Mother	11		47100222	5022242
TRAILS	Mother	13		47100222	5023877
TRAILS	Mother	16		47100222	5021904
TRAILS	Teacher	11		47100222	5023980
TRAILS	Teacher	13		47100222	4051171
TRAILS	Teacher	16		47100222	4059150
VTSABD	Self	13	CAPA*	7991599	4080551
VTSABD	Self	16	CAPA*	7724021	3950632
VTSABD	Self	13	MA*	7950086	4080551
VTSABD	Self	16	MA*	7972919	4080551
VTSABD	Parent	13	CAPA*	7993832	4080551
VTSABD	Parent	16	CAPA*	7960825	4080551
VTSABD	Parent	13	Rutter*	7953270	4080551
VTSABD	Parent	16	Rutter*	7988403	4080551
YFS	Mother	wide		15077966	5128429

* = measurement instrument (described in detail in Supplementary Table 3)

Table S8 Stratified analyses of internalising symptoms

N = number; N_{unique} = number of unique individuals; N_{obs} = total number of observations; N_{eff} = effective sample size; h^2 = SNP-based heritability; SE = standard error, CI = confidence interval

Category	Meta-analysis	Age range		N					LDSC-statistics						
		min	max	Cohorts	GWASs	N_{unique}	N_{obs}	N_{eff}	h^2	SE	Lower CI	Upper CI	Z	Intercept	SE
Overall	INToverall	3	18	22	125	64641	251152	132260	0.0166	0.0042	0.0084	0.0248	3.9524	1.0426	0.0075
Rater	Parent	3	18	20	69	50612	153856	82208	0.0172	0.0062	0.005	0.0294	2.7742	1.0373	0.0062
	Mother	3	16	10	35	34047	102194	56343	0.0256	0.0089	0.0082	0.043	2.8764	1.0452	0.0075
	Father	3	15	3	7	6323	17043	10494	0.0898	0.0461	-0.0006	0.1802	1.9479	0.9834	0.0078
	Teacher	7	16	8	23	22001	45944	37208	0.0365	0.013	0.011	0.062	2.8077	1.009	0.0075
	Self	9	16	11	31	29122	49366	37853	0.0563	0.013	0.0308	0.0818	4.3308	0.993	0.007
Age	Early childhood	3	6	6	13	21049	37870	27599	0.0093	0.0169	-0.0238	0.0424	0.5503	1.0082	0.0065
	Mid-childhood	7	10	14	40	37296	90291	62630	0.0138	0.0076	-0.0011	0.0287	1.8158	1.0209	0.0076
	Late childhood	11	12	10	24	23311	46120	35162	0.0064	0.0145	-0.022	0.0348	0.4414	1.0263	0.0071
	Adolescence	13	18	16	47	33201	75263	58502	0.0197	0.0085	0.003	0.0364	2.3176	1.0638	0.0068
Rater-by-age	Parent, early childhood	3	6	6	13	21049	37870	28724	0.0157	0.0177	-0.019	0.0504	0.887	1.0061	0.0068
	Parent, mid-childhood	7	10	13	26	34734	60654	43511	0.0206	0.0108	-0.0006	0.0418	1.9074	1.0107	0.0066
	Parent, late-childhood	11	12	8	11	21383	23982	21950	-0.0182	0.0214	-0.0601	0.0237	-0.8505	1.0203	0.007
	Parent, adolescence	13	16	9	18	20623	29742	25182	0.0269	0.0199	-0.0121	0.0659	1.3518	1.0339	0.0068
	Mother, early childhood	3	6	5	9	19401	29219	24061	0.0049	0.0195	-0.0333	0.0431	0.2513	2.008	0.0075
	Mother, mid-childhood	7	10	7	10	21270	35046	27337	0.0314	0.0167	-0.0013	0.0641	1.8802	1.0086	0.0071
	Mother, adolescence	13	16	5	8	16315	21094	19700	0.0154	0.0259	-0.0354	0.0662	0.5946	1.051	0.0074
	Teacher, mid-childhood	7	10	7	10	19317	27001	24766	0.0241	0.0192	-0.0135	0.0617	1.2552	1.0161	0.0069
	Self, adolescence	13	18	11	23	26777	37573	31496	0.032	0.0146	0.0034	0.0606	2.1918	0.9981	0.0068
Instrument	ASEBA	3	18	10	47	25892	91093	43442	0.0311	0.013	0.0056	0.0566	2.3923	1.0068	0.0078
	SDQ	3	16	5	33	21396	96010	50880	0.0262	0.0116	0.0035	0.0489	2.2586	1.0861	0.0086
	Rutter	7	16	3	11	7654	20562	13997	0.0037	0.0336	-0.0622	0.0696	0.1101	0.9912	0.0068

Table S9 List of external phenotypes for genetic correlations

Domain	Trait	Child/Adult/Both	Paper	Digital object identifier (doi)	Source
Psychiatry	Anorexia Nervosa	Both	Watson, 2019	https://doi.org/10.1038/s41588-019-0439-2	https://www.med.unc.edu/pgc/download-results/ed/
Psychiatry	Anxiety	Adult	Purves, 2019	https://doi.org/10.1038/s41380-019-0559-1	Authors
Psychiatry	Attention deficit hyperactivity disorder (ADHD)	Both	Demontis, 2019	https://doi.org/10.1038/s41588-018-0269-7	https://ipsych.dk/en/research/downloads/
Psychiatry	Autism spectrum disorder	Both	Grove, 2019	https://doi.org/10.1038/s41588-019-0344-8	https://www.med.unc.edu/pgc/results-and-downloads/
Psychiatry	Bipolar disorder	Adult	Stahl, 2018	https://doi.org/10.1101/173062	https://www.med.unc.edu/pgc/results-and-downloads/
Psychiatry	Depressive symptoms	Adult	Baselmans, 2019	https://doi.org/10.1038/s41588-018-0320-8	https://surfdrive.surf.nl/files/index.php/s/Ow1qCDpFT421ZOO/download?path=%2FMultivariate_GWAMA_sumstats%2FMA_GWAMA&files=MA_DEPno23andMe_18022020.txt.gz
Psychiatry	Major depressive disorder	Adult	Howard, 2019	https://doi.org/10.1038/s41593-018-0326-7	https://www.med.unc.edu/pgc/results-and-downloads/
Psychiatry	Obsessive-compulsive disorder	Both	Arnold, 2018	https://doi.org/10.1038/mp.2017.154	https://www.med.unc.edu/pgc/results-and-downloads/
Psychiatry	Schizophrenia	Adult	Pardiñas, 2018	https://doi.org/10.1038/s41588-018-0059-2	https://walters.psycm.cf.ac.uk/clozuk_pgc2.meta.sumstats.txt.gz
Psychological	Childhood aggression, overall	Child	Ip, 2019	https://doi.org/10.1101/854927	Authors
Psychological	Childhood aggression, maternal report	Child	Ip, 2019	https://doi.org/10.1101/854927	Authors
Psychological	Childhood aggression, teacher report	Child	Ip, 2019	https://doi.org/10.1101/854927	Authors
Psychological	Childhood aggression, self report	Child	Ip, 2019	https://doi.org/10.1101/854927	Authors
Psychological	Loneliness	Adult	Day, 2018	https://doi.org/10.1038/s41467-018-04930-1	https://www.repository.cam.ac.uk/bitstream/handle/1810/277812/Day_2018_NatComs_Social.tar.gz?sequence=1&isAllowed=y
Psychological	Neuroticism	Adult	Nagel, 2018	https://doi.org/10.1038/s41588-018-0151-7	https://ctg.cncr.nl/documents/p1651/sumstats_neuroticism_ctg_format.txt.gz
Psychological	Well-being spectrum	Adult	Baselmans, 2019	https://doi.org/10.1038/s41588-018-0320-8	https://surfdrive.surf.nl/files/index.php/s/Ow1qCDpFT421ZOO/download?path=%2F&files=N_GWAMA_WBspectrum_no23andME.txt_04072019.txt.gz
Psychological	Insomnia	Adult	Jansen, 2019	https://doi.org/10.1038/s41588-018-0333-3	https://ctg.cncr.nl/documents/p1651/Insomnia_sumstats_Jansenetal.txt.gz
Physical health	Coronary artery disease	Adult	Nikpay, 2019	https://doi.org/10.1038/ng.3396	http://www.cardiogramplusc4d.org/media/cardiogramplusc4d-consortium/data-downloads/cad.additive.Oct2015.pub.zip
Reproduction	Age at menarche	Adult	Day, 2017	https://doi.org/10.1038/ng.3841	https://www.reprogen.org/Menarche_1KG_NatGen2017_WebsiteUpload.zip
Reproduction	Age at first birth	Adult	Barban, 2016	https://doi.org/10.1038/ng.3698	http://sociogenome.com/material/GWASresults/AgeFirstBirth_Pooled.txt.gz
Substance use	Smoking initiation	Both	Liu, 2019	https://doi.org/10.1038/s41588-018-0307-5	https://conservancy.umn.edu/bitstream/handle/11299/201564/SmokingInitiation.txt.gz?sequence=27&isAllowed=y
Substance use	Cigarettes per day	Both	Liu, 2019	https://doi.org/10.1038/s41588-018-0307-5	https://conservancy.umn.edu/bitstream/handle/11299/201564/CigarettesPerDay.txt.gz?sequence=17&isAllowed=y
Substance use	Drinks per week	Both	Liu, 2019	https://doi.org/10.1038/s41588-018-0307-5	https://conservancy.umn.edu/bitstream/handle/11299/201564/DrinksPerWeek.txt.gz?sequence=18&isAllowed=y
Cognition	Educational attainment	Adult	Lee, 2018	https://doi.org/10.1038/s41588-018-0147-3	https://www.dropbox.com/s/ho58e9jmytmpaf8/GWAS_EA_excl23andMe.txt?dl=0
Cognition	Intelligence	Both	Savage, 2018	https://doi.org/10.1038/s41588-018-0152-6	https://ctg.cncr.nl/documents/p1651/SavageJansen_IntMeta_sumstats.zip
Anthropometric	Body-mass index (BMI)	Adult	Yengo, 2018	https://doi.org/10.1093/hmg/ddy271	http://cnsgenomics.com/data/yengo_et_al_2018_hmg/Meta-analysis_Locke_et_al+UKBiobank_2018_UPDATED.txt.gz
Anthropometric	Height	Adult	Yengo, 2018	https://doi.org/10.1093/hmg/ddy271	http://cnsgenomics.com/data/yengo_et_al_2018_hmg/Meta-analysis_Wood_et_al+UKBiobank_2018.txt.gz

Table S10 Top 10 genes from MAGMA gene-based test
CHR = chromosome; START = first base pair position; END = last base pair position; NSNPS = number of included SNPs; NPARAM = the number of relevant parameters used in the model; N =sample size; ZSTAT = Z-score of the association; P = p-value

GENE	CHR	START	STOP	NSNPS	NPARAM	N	ZSTAT	P
CCL26	7	75398851	75419214	68	6	91224	4.3624	6.43E-06
ARHGAP27	17	43471275	43511787	94	6	105289	4.3219	7.73E-06
CENPO	2	25016005	25045245	53	7	121977	4.2089	1.28E-05
RNF17	13	25338290	25454059	288	14	125625	4.2004	1.33E-05
PLEKHM1	17	43513266	43568115	78	4	102143	4.1419	1.72E-05
PTRHD1	2	25012603	25016251	9	3	127234	4.1133	1.95E-05
CENPJ	13	25457171	25497018	90	12	125402	3.9077	4.66E-05
FAM73A	1	78245309	78344106	51	7	112008	3.7949	7.39E-05
WNT3	17	44839872	44910520	90	13	101004	3.6071	1.55E-04
FAM162B	6	117073363	117086886	33	6	115633	3.5847	1.69E-04

Table S11 MAGMA tissue expression analyses
SE = standard error; P = p-value

30 broad tissue types

VARIABLE	BETA	SE	P
Brain	0.015076	0.0066204	0.011395
Stomach	0.020195	0.013163	0.062495
Pituitary	0.0093882	0.0079843	0.11984
Colon	0.017582	0.01543	0.12727
Blood_Vessel	0.011474	0.011324	0.15547
Adipose_Tissue	0.012417	0.012882	0.16757
Testis	0.005178	0.0054295	0.17013
Blood	0.0043934	0.0060019	0.23209
Breast	0.01081	0.015224	0.23884
Nerve	0.007002	0.0099397	0.24058
Uterus	0.0062439	0.011603	0.29524
Esophagus	0.0088292	0.01671	0.29862
Ovary	0.0019138	0.0099615	0.42383
Liver	-0.00013442	0.0063287	0.50847
Salivary_Gland	-0.00080142	0.010432	0.53062
Muscle	-0.0018264	0.0067093	0.60727
Fallopian_Tube	-0.0039227	0.013497	0.61433
Bladder	-0.0048272	0.015741	0.62045
Skin	-0.0036457	0.0097597	0.64563
Cervix_Uteri	-0.0056547	0.013728	0.65979
Vagina	-0.0064647	0.012352	0.69964
Pancreas	-0.005696	0.0084993	0.74862
Spleen	-0.0054981	0.0076198	0.76471
Lung	-0.0080747	0.010174	0.78629
Kidney	-0.0086463	0.0091343	0.82806
Adrenal_Gland	-0.014411	0.010095	0.92327
Small_Intestine	-0.015483	0.010505	0.92972
Prostate	-0.022928	0.013405	0.9564
Heart	-0.016299	0.0092995	0.96016
Thyroid	-0.022236	0.01048	0.98306

53 specific tissue types

VARIABLE	BETA	SE	P
Brain_Anterior_cingulate_cortex_BA24	0.018651	0.0069528	0.0036567
Brain_Frontal_Cortex_BA9	0.016635	0.0066165	0.0059698
Brain_Cortex	0.01694	0.0068568	0.0067513
Brain_Amygdala	0.017202	0.0075484	0.011345
Brain_Hypothalamus	0.015908	0.0078102	0.020843
Brain_Nucleus_accumbens_basal_ganglia	0.014838	0.0073132	0.021243
Brain_Cerebellum	0.012165	0.0060643	0.02244
Brain_Cerebellar_Hemisphere	0.011349	0.0058849	0.026906
Brain_Hippocampus	0.012705	0.0076773	0.048988
Brain_Caudate_basal_ganglia	0.010792	0.0075715	0.077034
Brain_Putamen_basal_ganglia	0.010145	0.007634	0.091945
Brain_Spinal_cord_cervical_c-1	0.0092582	0.0083017	0.13239
Brain_Substantia_nigra	0.0080862	0.0082645	0.16394
Testis	0.0036998	0.0054236	0.24757
Pituitary	0.0054668	0.0085721	0.26183
Whole_Blood	0.0031159	0.0054488	0.28372
Stomach	0.0058197	0.011537	0.30699
Artery_Tibial	0.004642	0.0096294	0.31488
Colon_Sigmoid	0.0025874	0.012134	0.41557
Esophagus_Muscularis	0.0013069	0.012026	0.45673
Adipose_Subcutaneous	0.00088892	0.0103	0.46561
Esophagus_Gastroesophageal_Junction	-0.000784	0.012402	0.5252
Nerve_Tibial	-0.0008784	0.0096087	0.53642
Adipose_Visceral_Omentum	-0.002286	0.010708	0.58452
Artery_Aorta	-0.0021257	0.0098409	0.58551
Colon_Transverse	-0.0027239	0.011182	0.59623
Breast_Mammary_Tissue	-0.0045121	0.012093	0.64547
Uterus	-0.0039259	0.010513	0.64558
Liver	-0.0024463	0.0061166	0.6554
Cells_Cultured_fibroblasts	-0.0035796	0.0063728	0.71283
Cells_EBV-transformed_lymphocytes	-0.0026727	0.0047083	0.71486
Ovary	-0.0053277	0.0093426	0.71575
Artery_Coronary	-0.0078348	0.011418	0.7537
Minor_Salivary_Gland	-0.0067584	0.0092147	0.76835
Muscle_Skeletal	-0.0050746	0.0067316	0.77452
Skin_Sun_Exposed_Lower_leg	-0.0067165	0.0074428	0.81658
Esophagus_Mucosa	-0.0067249	0.0073252	0.8207
Skin_Not_Sun_Exposed_Suprapubic	-0.0068849	0.0074223	0.82318
Cervix_Ectocervix	-0.013104	0.011698	0.86869
Fallopian_Tube	-0.012932	0.011436	0.87093
Pancreas	-0.009344	0.0081166	0.87517
Spleen	-0.008381	0.0070843	0.8816
Bladder	-0.01589	0.012693	0.89468
Vagina	-0.013492	0.010666	0.89704
Lung	-0.011759	0.008799	0.90928
Cervix_Endocervix	-0.015008	0.011121	0.91141
Kidney_Cortex	-0.012667	0.0086335	0.92883
Kidney_Medulla	-0.015263	0.0088533	0.95764
Heart_Left_Ventricle	-0.017038	0.008801	0.97355
Small_Intestine_Terminal_Ileum	-0.018118	0.0092724	0.97464
Adrenal_Gland	-0.020882	0.0097723	0.98369
Prostate	-0.026999	0.01153	0.99039
Thyroid	-0.024534	0.0093667	0.99559
Heart_Atrial_Appendage	-0.025181	0.0092355	0.9968

Table S12 Top 10 gene sets from MAGMA gene-set tissue analysis
N genes = number of genes; STD = standardised; SE = standard error; P = p-value

Gene set	N genes	Beta	Beta STD	SE	P
GO_cc:go_translation_preinitiation_complex	15	0.70132	0.019913	0.19642	0.00017861
Curated_gene_sets:rickman_head_and_neck_cancer_d	33	0.52195	0.021971	0.14685	0.00019003
Curated_gene_sets:benporath_myc_targets_with_ebox	209	0.18605	0.019616	0.05377	0.0002706
GO_bp:go_gmp_metabolic_process	8	0.89822	0.018629	0.26308	0.00032051
Curated_gene_sets:ren_alveolar_rhabdomyosarcoma_up	95	0.28963	0.020651	0.085015	0.00032946
GO_bp:go_cytoplasmic_translational_initiation	27	0.49469	0.018839	0.1454	0.00033495
GO_cc:go_mcm_complex	11	0.67667	0.016455	0.20034	0.00036637
GO_bp:go_positive_regulation_of_receptor_binding	9	0.8465	0.01862	0.25176	0.00038731
GO_bp:go_formation_of_cytoplasmic_translation_initiation_complex	12	0.69833	0.017736	0.21086	0.00046443
GO_mf:go_guanylate_kinase_activity	7	0.98205	0.019052	0.29755	0.0004837

Table S13 Genetic correlations between internalising symptoms and external phenotypes, ordered by the strength of the correlation with $INT_{overall}$
r_g= genetic correlation; se = standard error of the genetic correlation; p = p-value
Results in bold indicate genetic correlations that are statistically different from zero after Bonferroni correction for 2 x 27=54 tests; *p* < 9.26E-04

		$INT_{overall}$			INT_{self}		
Domain	Trait	r_g	se	p	r_g	se	p
Psychological	Well-being spectrum	**-0.7861**	**0.0966**	**4.01E-16**	**-0.6339**	**0.0806**	**3.59E-15**
Psychiatry	Anxiety	**0.7612**	**0.1303**	**5.10E-09**	**0.4564**	**0.1062**	**1.73E-05**
Psychological	Neuroticism	**0.7581**	**0.1015**	**8.32E-14**	**0.6585**	**0.0846**	**6.86E-15**
Psychiatry	Major depressive disorder	**0.7162**	**0.0978**	**2.43E-13**	**0.6006**	**0.0881**	**9.38E-12**
Psychiatry	Depressive symptoms*	**0.6981**	**0.0887**	**3.65E-15**	**0.5959**	**0.0825**	**4.95E-13**
Psychological	Loneliness	**0.6019**	**0.0956**	**3.10E-10**	**0.5913**	**0.0871**	**1.15E-11**
Psychological	Childhood aggression, maternal report	**0.5931**	**0.1117**	**1.11E-07**	0.1898	0.1406	0.1771
Psychological	Childhood aggression, overall	**0.5778**	**0.1075**	**7.74E-08**	0.3132	0.1236	0.0113
Psychiatry	Attention deficit hyperactivity disorder (ADHD)	**0.5614**	**0.1165**	**1.43E-06**	**0.4874**	**0.101**	**1.40E-06**
Psychiatry	Autism spectrum disorder	**0.5114**	**0.1193**	**1.82E-05**	0.4014	0.1216	0.001
Psychological	Insomnia	**0.4234**	**0.0813**	**1.89E-07**	**0.2798**	**0.077**	**0.0003**
Reproduction	Age at first birth	**-0.3588**	**0.0989**	**0.0003**	-0.2164	0.0875	0.0134
Cognition	Educational attainment	**-0.3481**	**0.0652**	**9.20E-08**	**-0.222**	**0.0587**	**0.0002**
Substance use	Cigarettes per day	**-0.3398**	**0.0884**	**0.0001**	**-0.3194**	**0.0764**	**2.87E-05**
Psychological	Childhood aggression, self report	0.3336	0.1494	0.0256	**0.6612**	**0.1513**	**1.23E-05**
Cognition	Intelligence	**-0.3255**	**0.0733**	**9.04E-06**	-0.1271	0.0616	0.0392
Psychiatry	Schizophrenia	0.2031	0.0671	0.0025	0.1439	0.0655	0.0281
Substance use	Drinks per week	0.192	0.076	0.0115	0.0038	0.0658	0.9538
Anthropometric	Body-mass index (BMI)	0.1738	0.0599	0.0037	**0.2069**	**0.0592**	**0.0005**
Anthropometric	Height	-0.1532	0.0468	0.0011	-0.0985	0.0477	0.0391
Psychiatry	Bipolar disorder	0.0779	0.0794	0.3266	0.0216	0.0784	0.7833
Reproduction	Age at menarche	-0.067	0.0583	0.2508	-0.1561	0.0524	0.0029
Physical health	Coronary artery disease	0.0524	0.0774	0.4983	0.0767	0.0805	0.3407
Substance use	Smoking initiation	-0.0494	0.0542	0.3616	**-0.211**	**0.0573**	**0.0002**
Psychiatry	Obsessive-compulsive disorder	-0.0247	0.1586	0.8765	-0.1583	0.1455	0.2765
Psychiatry	Anorexia Nervosa	-0.0196	0.0839	0.8148	0.0727	0.0815	0.3725
Psychological	Childhood aggression, teacher report	-0.015	0.1521	0.9216	0.0964	0.1707	0.572

*= depressive symptoms were reverse-coded in the GWAS, so that higher score meant fewer depressive symptoms. Subsequently, the SNP effects were reversed for the calculation of rG

Table S14 Polygenic score analyses

M7 = maternal-report at age 7; S13to18 = self-report during adolescence; N = sample size; se = standard error; p = p-value; r^2 = percentage of variance explained

Bold p-values indicate scores with significant beta's after Bonferroni correction for 2 tests; $p < 0.025$

Predictor - PGS	Outcome - phenotype	N	Prior	beta	se	p	r^2
INT overall	Internalising, M7	3845	0.5	0.06180371	0.0182518	**0.000708752**	0.3819699
INT overall	Internalising, S13to18	2679	0.5	0.01759981	0.02006937	0.3805144	0.03097534
